# Fixation versus periphery in visual awareness: Differential effects of recent perceptual experience

**DOI:** 10.1167/jov.25.7.2

**Published:** 2025-06-03

**Authors:** Tim Gastrell, Matt Oxner, Frank Schumann, David Carmel

**Affiliations:** 1 School of Psychology, Victoria University of Wellington, Wellington, New Zealand; 2 Queensland Brain Institute, The University of Queensland, Brisbane, Australia; 3 Wilhelm Wundt Institute for Psychology, University of Leipzig, Leipzig, Germany; 4 Laboratoire des systèmes perceptifs, Département d’études cognitives, École normale supérieure, Université PSL, CNRS, Paris, France; 5 Université de Paris, CNRS, Integrative Neuroscience & Cognition Center, Paris, France; 6 Sorbonne Université, INSERM, CNRS, Institut de la Vision, Paris, France

**Keywords:** visual awareness, priming, structure from motion, fixation versus periphery

## Abstract

Processing differences between foveal and peripheral vision mean that the location of objects in the visual field can strongly influence the way we experience them. The contents of visual awareness are believed to arise from interactions between sensory stimulation and context (e.g., expectations formed by recent experience), but the effect of visual field location on these interactions remains unclear. Here, we compared the effects of recent experience on awareness at fixation versus the periphery. On each trial, observers saw a brief display of an unambiguously rotating structure-from-motion prime sphere, followed by a brief display of a probe sphere with ambiguous motion. Experiment 1 established that conscious perception of the motion direction of the probe was more likely to differ from the prime when the stimuli were presented in the periphery compared with fixation. Experiment 2 ruled out a high-level, non-retinotopic, precision-weighting account of this effect by demonstrating that, although priming was apparent when the stimulus moved from fixation to periphery or vice versa, its magnitude was the same for low-precision peripheral and high-precision fixated primes. Experiment 3 replicated the original location effect and also found stronger motion adaptation in the periphery; the effects were not correlated, though, indicating that motion adaptation cannot account for the location effect. Experiment 4 replicated the location effect again and ruled out differences in fixation stability as the underlying mechanism. Overall, our results demonstrate a robust effect of visual field location on the integration of recent visual experience during construction of perceptual awareness and highlight the need to elucidate the mechanisms underlying differential generation of visual experience across the visual field.

## Introduction

Our perceptual experience of objects in the environment can vary considerably depending on where in the visual field they are located. Objects presented to foveal vision are crisp, detailed and easily discernible; conversely, stimuli shown in the periphery, where the information available to the visual system is sparser, are more difficult to discern. Such differences in visual experience stem from fundamental variations in the structural and functional properties of the early visual system at different retinal locations and their retinotopic representations. These variations range from differences in the ratio of rod to cone receptors in the retina (low in the fovea and high in the periphery) to the size and density of neuronal receptive fields in the visual cortex (from small and dense in the fovea to large and sparse in the periphery). Functionally, it is well established that, compared with fixation, both detection and discrimination of visual stimuli diminish significantly in the periphery (Duncan & Boynton, 2003), as do detection and discrimination of motion (Levi, Klein, & Aitsebaomo, 1984; Mckee & Nakayama, 1984; Raymond, 1994).

Visual awareness—the conscious experience associated with a visual percept—is broadly believed to arise from interactions between sensory stimulation (the information currently available to the visual system) and context (e.g., prior visual experience and expectations) (de Lange, Heilbron, & Kok, 2018; Hohwy, Roepstorff, & Friston, 2008). The perceptual heterogeneity between fixation and periphery affects the processing of sensory stimulation and may also affect the integration of context, but investigations into the mechanisms of visual awareness have focused primarily on observers’ experiences of fixated stimuli (for a review, see Brascamp, Sterzer, Blake, & Knapen, 2018). Comparatively few studies have asked how the dynamics of awareness might differ between fixated and peripheral visual field locations (Breitmeyer & Ritter, 1986; Rutherford, 2003). Although this disparity in the literature may not be surprising given the prominence of foveal vision in normal viewing of visual scenes, a full understanding of visual awareness requires an examination of how it arises at different locations in the visual field. Here, we address this gap by comparing the effect of expectations created by immediate perceptual history (priming) on conscious perception of bistable stimuli at fixation versus the periphery.

Bistable perception arises when the same stimulus is compatible with two perceptual interpretations; when viewed continuously, an unchanging bistable stimulus yields a pattern in which awareness alternates between those interpretations (Bareham, Oxner, Gastrell, & Carmel, 2020; Carmel, Arcaro, Kastner, & Hasson, 2010). Importantly, the percept that will *initially* dominate awareness may be influenced by both low-level attributes (e.g., contrast) (Blake & Logothetis, 2002) and high-level factors (e.g., expectation) (Denison, Piazza, & Silver, 2011; Sterzer, Frith, & Petrovic, 2008). To compare the influence of immediate perceptual history at fixation versus the periphery, we measured the effect, at each location, of an unambiguous prime stimulus on the initial percept of a subsequent bistable stimulus.

We used structure-from-motion (SFM) stimuli, which consisted of multiple dots whose motion created the impression that they were placed on the surface of a rotating sphere. In experimental trials, observers were first shown a prime stimulus—a sphere with a defined, unambiguous rotation direction. This was followed by a probe—a similar but ambiguous stimulus that could be seen as rotating either in the same direction as the prime or in the opposite direction. We measured whether visual field location (fixation or periphery) would modulate the effect of the prime stimulus on the initial perception of the probe stimulus.

We hypothesized, straightforwardly, that the priming effect of the unambiguous stimulus on awareness of an ambiguous probe would be smaller in the periphery than at fixation. Different underlying mechanisms may independently give rise to such an effect: First, it would be in line with increasingly popular predictive coding accounts of bistable vision (e.g., Hohwy et al., 2008), which suggest that conscious percepts are drawn from Bayesian posterior distributions, constructed as the precision-weighted average of sensory priors (i.e., perceptual history) and likelihoods (i.e., sensory evidence). For bistable stimuli, the sensory evidence is equally likely to be consistent with either percept; an unambiguous prime is consistent with one of the percepts, generating a prior representation of the expected stimulus; and precision-weighted averaging of the two therefore leads to a posterior that is biased toward the prime. Crucially, both the priors (the perceptual history provided by the prime) and the likelihoods (the sensory evidence provided by the probe) are encoded with lower precision when they are presented in the periphery of the visual field than when they are shown at fixation; therefore, compared with presentations at fixation, if both the prime and probe are presented in the periphery then each of them contributes lower precision to the precision weighting that generates the posterior distribution, yielding a less precise posterior distribution that contains a greater proportion of potential percepts that are dissimilar to the prime (see Supplementary Section S1 and Supplementary Figure S1 for a more detailed explanation of this account). Under this framework, peripheral viewing of primes should diminish their influence compared with fixated primes.

This predictive coding view relies on the notion that awareness is driven by top–down predictions produced by a generative model of the most probable causes of sensory events. This is an algorithmic description that can be implemented by a variety of mechanisms at various levels of processing. Recent work has increasingly focused on high-level predictive coding explanations for bistable vision (implemented beyond the point of retinotopic representation, such as computationally at the level of object representation or anatomically in frontoparietal cortex) (Bareham et al., 2020; Brascamp et al., 2018; Denison et al., 2011; Kanai, Carmel, Bahrami, & Rees, 2011; Weilnhammer, Stuke, Hesselmann, Sterzer, & Schmack, 2017; Weilnhammer et al., 2021). However, predictive coding accounts can apply to low-level retinotopic mechanisms as well, and low-level mechanisms such as neuronal adaptation and inhibition have also been proposed to account for switches in bistable perception (Blake & Logothetis, 2002; Chen & He, 2004). Low-level (retinotopic) neural adaptation can indeed provide an account for diminished priming of a bistable percept in the periphery: An unambiguous SFM prime may cause motion adaptation, leading to a subsequent ambiguous stimulus being perceived as moving in the opposite direction. According to this mechanism, the prime generates *more* effective adaptation in the periphery, in contrast to the *less* precise representation of unambiguous motion direction in the high-level account, but (critically) it yields the same prediction: If motion adaptation in the periphery is greater than at fixation, it will lead to a greater proportion of perceptual switches between prime and probe in the periphery than at fixation.

These different mechanisms are not mutually exclusive nor are they exhaustive; multiple high-level and low-level processes may influence differences in awareness at fixation versus the periphery. For example, Pastukhov & Braun (2013) demonstrated evidence for at least two types of priming effects of perceptual history on bistable vision, each of which operates on a different timescale: a neural persistence effect that decays rapidly, and a perceptual memory effect that arises and decays slowly. In our experiments, the transition from unambiguous prime to ambiguous stimulus was either immediate or followed a brief blank period; therefore, the relevant mechanism is likely to be a short-term persistence of previous neural representations.

The four experiments reported below explore some of these possible influences: First, in Experiment 1, we established that observers are indeed more likely to report switches in rotation direction between prime and probe stimuli at a peripheral location compared with fixation. In Experiment 2, we found that this difference cannot be attributed to high-level, non-retinotopic representations of the stimuli, casting doubt on a predictive coding account of the effect at the level of object representation. Experiment 3 replicated the original effect of reduced priming in the periphery; it also revealed greater motion adaptation in the periphery, but this adaptation is not associated with the increased switch probabilities in peripheral vision and is thus unlikely to explain it. Finally, Experiment 4 again replicated the effect of greater switch probabilities in the periphery compared with fixation while ruling out differences in fixational eye movements as a potential explanation for the effect.

## Experiment 1

In our first experiment, we asked whether the location of the display—at fixation or in the periphery—would modulate the effect of an unambiguous prime on observers’ perceptual awareness of an ambiguous SFM animation. To test this, participants were primed with a brief (1000 ms) disambiguated rotating sphere, which then became ambiguous (for 700 ms). We measured the effect of the prime as the probability of reporting a switch in perceived rotation between the unambiguous prime and the ambiguous probe. As described above, we predicted that switch rates would be higher when the prime and probe spheres were presented in the periphery compared with fixation. An online version of this experiment, with similar methods and identical analyses, was preregistered on the Open Science Framework (https://osf.io/9r37t); due to poor-quality online eye-tracking data, we abandoned that study and ran the present experiment when COVID-19 restrictions on lab-based work were removed.

### Method

#### Participants

Fifty-two undergraduate psychology students and participants from the community participated voluntarily for course credit or supermarket vouchers. Of this sample, 11 participants were excluded for their performance on catch trials (see Design section below); none were excluded for moving their eyes too often (see Results section for exclusion criteria). This led to a final sample of 41 participants (12 identified as male, 28 as female, and one as non-binary; mean age, 20.20 ± 3.37 years; by self-report, 36 were right handed, four were left handed, and one was ambidextrous). We chose this sample size based on a power analysis (using G*Power) (Erdfelder, Faul, Buchner, & Lang, 2009), which estimated a required sample of 41 to detect a moderate effect size (*d* = 0.4) at 80% power (α = 0.05). All participants across all four experiments had normal or corrected-to-normal vision and provided written informed consent before participating. All four experiments were approved by the Victoria University of Wellington Human Ethics Committee which adheres to the tenets of the Declaration of Helsinki.

#### Stimuli

Ambiguous SFM spheres were composed of 300 gray dots, each with 0.039 degree of visual angle (dva) radius and 146.52 cd/m^2^ luminance, scattered randomly across a circular area (radius, 1.0 dva) against a black background (0.44 cd/m^2^ luminance). The motion of the dots followed a sinusoidal speed profile, such that they mimicked a flat projection of dots scattered on a transparent globe rotating around a central axis with an angular velocity of 51.7° per second (De Graaf, De Jong, Goebel, Van Ee, & Sack, 2011). The rotation axis always lay on the plane of the screen (i.e., perpendicular to the observer). Dot positions were updated at 30 frames per second.

We generated unambiguous SFM spheres using an identical animation but with an added luminance disparity between the component dots that traveled in opposite directions; dots traveling in one direction were considerably brighter (267.65 cd/m^2^ luminance) than dots traveling in the opposite direction (30.62 cd/m^2^ luminance). This disambiguated motion direction such that perception overwhelmingly conformed to the closer surface being composed of the brighter dots.

For ambiguous stimuli, individual biases toward specific rotation directions are maximal around cardinal axes (vertical and horizontal) (Wexler, Duyck, & Mamassian, 2015); to minimize such biases, spheres in this study rotated around one of four intercardinal axes (45°, 135°, 225°, or 315°), counterbalanced within each condition.

#### Apparatus

Participants were seated in a dimly lit room, using a chin rest placed 57 cm from the computer screen. Stimuli were displayed using gamma-corrected brightness values on a 27-inch flatscreen monitor (PG278QR, monitor gamma = 2.2; ASUS, Taipei, Taiwan) with a 2560 × 1440-pixel resolution and 60-Hz refresh rate. All stimuli were generated and presented using MATLAB (MathWorks, Natick, MA) with the Psychtoolbox 3 extension (Brainard, 1997; Pelli, 1997). Responses were made using a standard keyboard. Eye gaze was monitored in real time (sampling rate, 1000 Hz) using an EyeLink 1000 eye tracker (SR Research, Ottawa, ON, Canada) and the EyeLink Toolbox for MATLAB (Cornelissen, Peters, & Palmer, 2002).

#### Design

**Figure 1.**
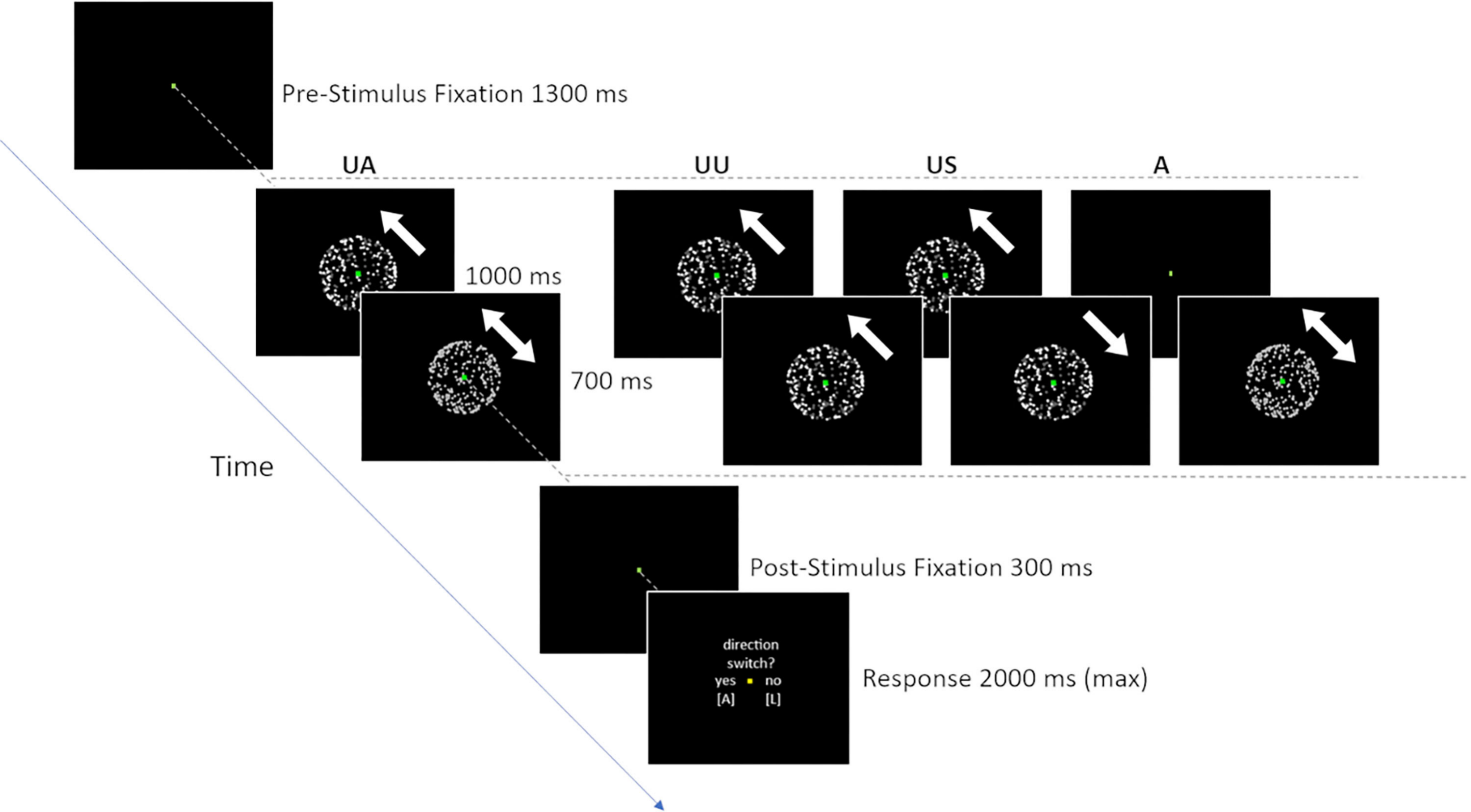
Schematic examples of a trial in different conditions presented at fixation. Peripheral trials were identical, except for the location of the spheres (5° left or right of fixation). Arrows indicate rotation direction: Unidirectional arrows indicate an unambiguous sphere and bidirectional arrows indicate an ambiguous sphere evoking a bistable percept. UA, unambiguous → ambiguous; UU, unambiguous → unambiguous (same direction); US, unambiguous → unambiguous (switch in direction); A, ambiguous only.

We manipulated the SFM animations in several ways to create the conditions described below (Figure 1) and compared reported perceptual effects for these conditions between the two visual field locations (fixated vs. peripheral). Stimulus conditions and visual field locations were manipulated within participants.

The primary trial type, henceforth referred to as the unambiguous–ambiguous (UA) condition, consisted of trials where an unambiguous prime sphere was presented for 1000 ms and then replaced by an ambiguous probe sphere presented for 700 ms. The rotation axis for the prime and probe spheres was maintained across the entire trial. Component dots were generated independently for the prime and probe phases.

After offset of the probe sphere, participants reported whether they had perceived a switch in rotation direction at any point during the display. We were interested in the effect of the unambiguous prime on the ambiguous probe—that is, whether the probe was first seen as rotating in the same direction as the prime or switched direction. Perceptual switches, however, may occur at other times during such presentations (e.g., they may happen spontaneously during the ambiguous probe phase, despite its short duration). To account for other switches that could occur during a trial, we included three additional conditions to collect baseline frequencies of reported switching during prime and probe phases, allowing us to control for the contribution of such switches to our results, as well as enabling us to monitor whether participants were attending to the task and able to perceive unambiguous stimuli.

Some switches reported in the UA condition may arise solely because the visual system adapts to the dominant, object-level representation of the unambiguous sphere. It is also possible, though unlikely, that switches may occur during the unambiguous sphere phase. (These spheres are not entirely unambiguous; it is possible, although much more difficult, to see the fainter dots as being on the closer surface of the sphere.) Although we considered it unlikely that either of these reasons would lead to a substantial proportion of switches in these brief displays, we included an unambiguous no-switch (UU) condition where an unambiguous prime sphere was presented for 1000 ms (as in the UA trials) and then replaced by a probe sphere with the same rotation axis that was unambiguous and rotated in the *same* direction for 700 ms. As expected, switches were indeed reported very rarely on these trials (see Results section). In line with our pre-registration, we therefore used them as one of two types of catch trial (see below).

We also included an unambiguous switch (US) condition, where an unambiguous prime sphere was presented for 1000 ms (as in UA trials) and then replaced by a probe sphere with the same rotation axis that was unambiguous and rotated in the *opposite* direction for 700 ms. These trials almost always led to a reported switch (see Results section) and provided a second type of catch trial. Catch trials of both kinds served the dual purpose of verifying that observers perceived the intended rotation direction of unambiguous sphere stimuli (as our predictions rely on observers being able to discern the prime) and that they were attending to the task (as there was a correct answer to the question of whether a switch had occurred: “no” in UU trials and “yes” in US trials).

Finally, a fully ambiguous (A) condition comprised trials where only an ambiguous SFM sphere was presented for 700 ms. These trials allowed us to measure baseline spontaneous switch rates for 700 ms presentations of ambiguous spheres. To equate the overall duration of trials in this condition with the other conditions, the pre-stimulus fixation dot that preceded these spheres was presented for an additional period of 1000 ms (the duration of the prime in the other trial types).

For each of the conditions (UA, UU, US, and A), half of all trials were presented at fixation, and half were presented 5.0° to the left or right (equally split).

#### Procedure

On each trial, participants viewed sphere animations belonging to one of the four trial types (UA, UU, US, or A) and then reported whether they had perceived a switch in the rotation direction of the spheres at any point during the trial. They were instructed to maintain their gaze on the central fixation point for the duration of each trial while blinking naturally throughout. Participants initially completed 10 practice trials, which demonstrated instances of each animation. Trials were presented randomly at one of the three possible locations, and participants were given the opportunity to clarify instructions with the experimenter.

Each trial began with a 1300 ms fixation point. Next, the sphere animation, comprising the prime and probe phases of the UA, UU, US, or A animations, was presented as described above. The animation was followed by another 300 ms fixation point. Participants were then prompted to press either the [A] keyboard key to indicate they had seen a switch in rotation direction, or the [L] key to indicate they had not. When a participant responded, the prompt disappeared, and the response period ended. If longer than 2000 ms elapsed without a response, the trial was terminated, and participants were instructed to respond faster via an on-screen message that lasted for 3000 ms. Similarly, if participants had not maintained their gaze on the fixation point during the trial, they were reminded not to move their eyes via another 3000 ms message that appeared after providing their response for that trial (such trials were excluded from analysis; see Results section). Trials were separated by a 500 ms intertrial interval during which the screen remained black.

Participants completed 144 UA trials (4 rotation angles × 2 levels of stimulus location [fixation or periphery] × 18 repetitions), and 48 of each of the UU, US, and A trials (4 rotation angles × 2 levels of stimulus location × 6 repetitions) totaling 288 trials. Trial order was randomized for each participant. Each session was divided into nine blocks of 32 trials; breaks between blocks were self-terminated. The eye tracker was recalibrated before each block. Participants spent 30 to 40 minutes completing the task, depending on response times and breaks, and they were debriefed upon completion.

#### Statistical analysis

We originally preregistered (and conducted) analyses that applied the general linear model (GLM) to the proportions of switch reports from each condition. However, we followed the advice of a reviewer who pointed out that binomial data such as our switch reports may violate GLM assumptions, particularly in near-ceiling/floor skewed distributions like those in some of our control conditions. Therefore, we instead fit logistic mixed models (estimated using maximum likelihood and Nelder–Mead optimizer) to individual trial responses in each stimulus condition to predict whether participants would report a switch as a function of eccentricity. The models included eccentricity as a fixed effect (formula : response ∼ eccentricity) and participant ID as a random effect (formula: ∼1|ID). One of our analyses used “baselined UA” data, accounting for estimated spontaneous switches based on the UU and A conditions (see Results section); as this is a composite measure, it was analyzed using switch proportions, for which we fit linear mixed models using the same effect structure as above. Full model outputs are available in Supplementary Section S5. Below we report test statistics and *p* values derived from these models using Wald approximations. We note that the results did not differ substantially in pattern or statistical significance between our preregistered and reported analyses. For simplicity of interpretation, the plots, reported Cohen's *dz* effect sizes, and associated confidence intervals (CIs) are derived from proportion data. The *p* values were not corrected for multiple comparisons unless otherwise stated, as we had a priori hypotheses for the fixated versus peripheral comparisons. Importantly, the UU, US, and A comparisons are control analyses for which larger effects would constitute evidence *against* our conclusion from the main (UA) analysis, so uncorrected *p* values provide a more conservative approach.

### Results

To validate our visual field manipulation, trials in which participants did not maintain fixation were excluded from analysis. Eye movements were determined by sampling horizontal gaze estimates recorded from the onset of the fixation point to the onset of the response screen. Trials were excluded if either the median or standard deviation of the horizontal deviation from the fixation point exceeded 2.0°. Trials in which participants did not respond were also excluded. Of the 288 trials collected from each participant, a mean of 8.1 trials were rejected (range, 0–56). We preregistered an exclusion criterion whereby participants for whom more than 33% of trials were rejected for these reasons would be excluded entirely (no participants met this criterion). Participants whose catch trial performance was below 80% correct were excluded from analysis because it was not clear that they were attending to the task (correct performance on catch trials consisted of reporting a switch on US trials and reporting no switch on UU).

Our primary dependent variable was participants’ binary yes/no responses for each trial, indicating whether they had perceived a switch in rotation direction. Preliminary analyses found no differences in switch probabilities between left and right peripheral trials or between the four rotation axes; peripheral trials were thus collapsed across presentation side, and each condition was collapsed across rotation axis (see Supplementary Section S2).

**Figure 2.**
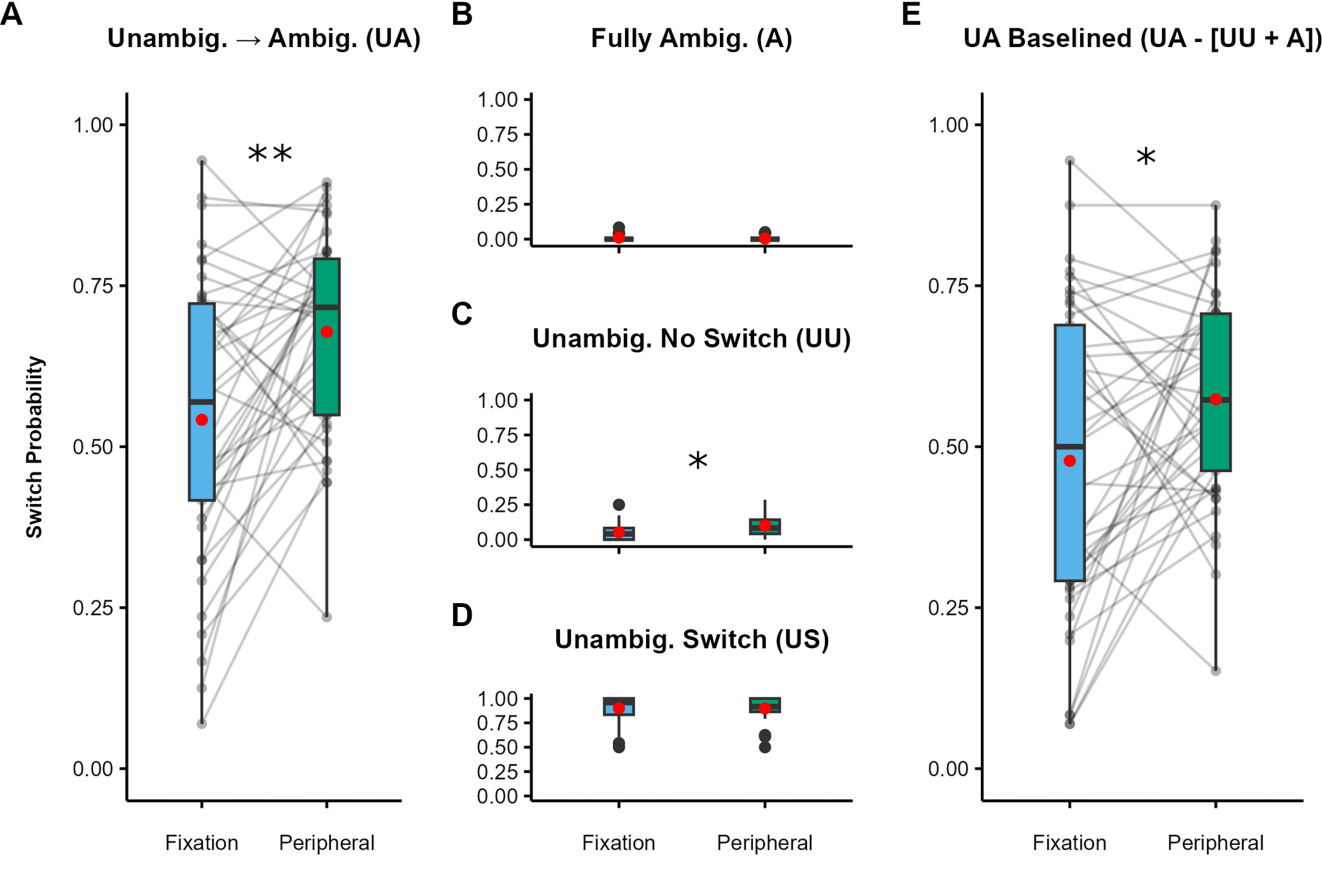
Experiment 1 results. (**A**–**D**) Switch probabilities for fixated and peripheral trials: UA condition (**A**), A condition (**B**), UU condition (**C**), and US condition (**D**). (**E**) UA trials baselined by subtracting the A and UU switch probabilities. Black dots indicate individual participants, red dots indicate condition means, boxes depict the interquartile range (IQR), and whiskers include all values < 1.5 × the IQR above or below the 75th or 25th percentile, respectively. *p < 0.05, **p < 0.001.

As predicted, the probability of reporting a switch on UA trials (Figure 2A) was significantly higher (*Z* = 11.27, *p* < 0.001) when the stimulus was presented in the periphery (*M* = 0.68) compared with when it was presented at fixation (*M* = 0.54; *dz* = 0.71; 95% CI, 0.30–1.12).

Reported switches in UA trials are extremely unlikely to reflect spontaneous switches during the ambiguous sphere phase alone: Switches in the A condition, where such spontaneous switches may occur, were rare (Figure 2B). Moreover, the difference in switch probability between fixated A trials (*M* = 0.01) and peripheral A trials (*M* = 0.004) was both non-significant (*Z* = –1.63, *p* = 0.104) and in the wrong direction to explain the effect of eccentricity observed in on UA trials.

Switches were also infrequent in the UU condition (Figure 2C). The probability of reporting a switch was significantly higher (*Z* = 3.90, *p* < 0.001) for peripheral UU trials (*M* = 0.10) compared with fixation trials (*M* = 0.05; *dz =* 0.65; 95% CI, 0.23–1.07). Notably, however, this difference (0.05) was substantially smaller than the effect observed in the UA condition (0.14), indicating that the effect in the UA trials cannot be attributed to object-level adaptation to the initial dominant percept.

As expected, switches were almost always reported in the US condition (Figure 2D). This was by design, as participants who repeatedly failed to report switches in this condition were excluded from analysis (all 11 participants excluded from analysis were removed because they had more than 20% errors on combined US and UU trials). There were no significant differences (*Z* = –0.12, *p* = 0.905) between fixated (*M =* 0.90) and peripheral (*M* = 0.90) trials.

As noted above, participants reported whether or not they had perceived a switch after stimulus offset and were instructed to report switches that had occurred at any point in the trial. Therefore, some reported switches in the UA condition may be due to spontaneous switching during the unambiguous phase (assessed in the A condition), as well as switches caused by adaptation to object-level representations of the unambiguous sphere (assessed in the UU condition). To control for the cumulative effect of such switches and estimate what proportion of switches was attributable to the transition from the unambiguous prime to the ambiguous probe, we calculated a baselined UA measure for fixated and peripheral trials by subtracting the switch rates of the A and UU conditions from the UA switch rate; that is, we employed the formula Baselined UA = UA – (A + UU). This baselined measure still showed significantly more switches (Figure 2E) on peripheral trials (*M* = 0.57) compared with fixated trials (*M* = 0.48), *t*(78) = 2.35, *p* *=* 0.022, *dz =* 0.46, 95% CI, 0.050–0.88, although the effect size (0.46) was smaller than for the non-baselined UA condition (0.71). Importantly, this result confirms that the effect of eccentricity observed in the UU condition does not fully explain the effect in the UA condition.

### Discussion

In this experiment, we aimed to test the prediction that, compared with fixation, peripheral viewing would reduce the effect of an unambiguous prime on perception of an ambiguous probe. In line with our prediction, participants were significantly more likely to report perceiving a switch in rotation at peripheral than fixated visual field locations (Figures 2A and 2E), demonstrating a spatial difference in the impact of perceptual history on percept selection.

This result is consistent with high-level predictive coding accounts of visual awareness in which the precision of predictions generated by non-retinotopic object representations is instrumental (Hohwy et al., 2008). However, it is also consistent with other candidate explanations. It is likely that at least some of the foveal–peripheral differences in the UA condition can be accounted for by other differences in early visual processing. In the following experiments, we investigated the extent to which the difference in switch rates can be accounted for by differences in high-level versus differences in low-level mechanisms.

## Experiment 2

**Figure 3.**
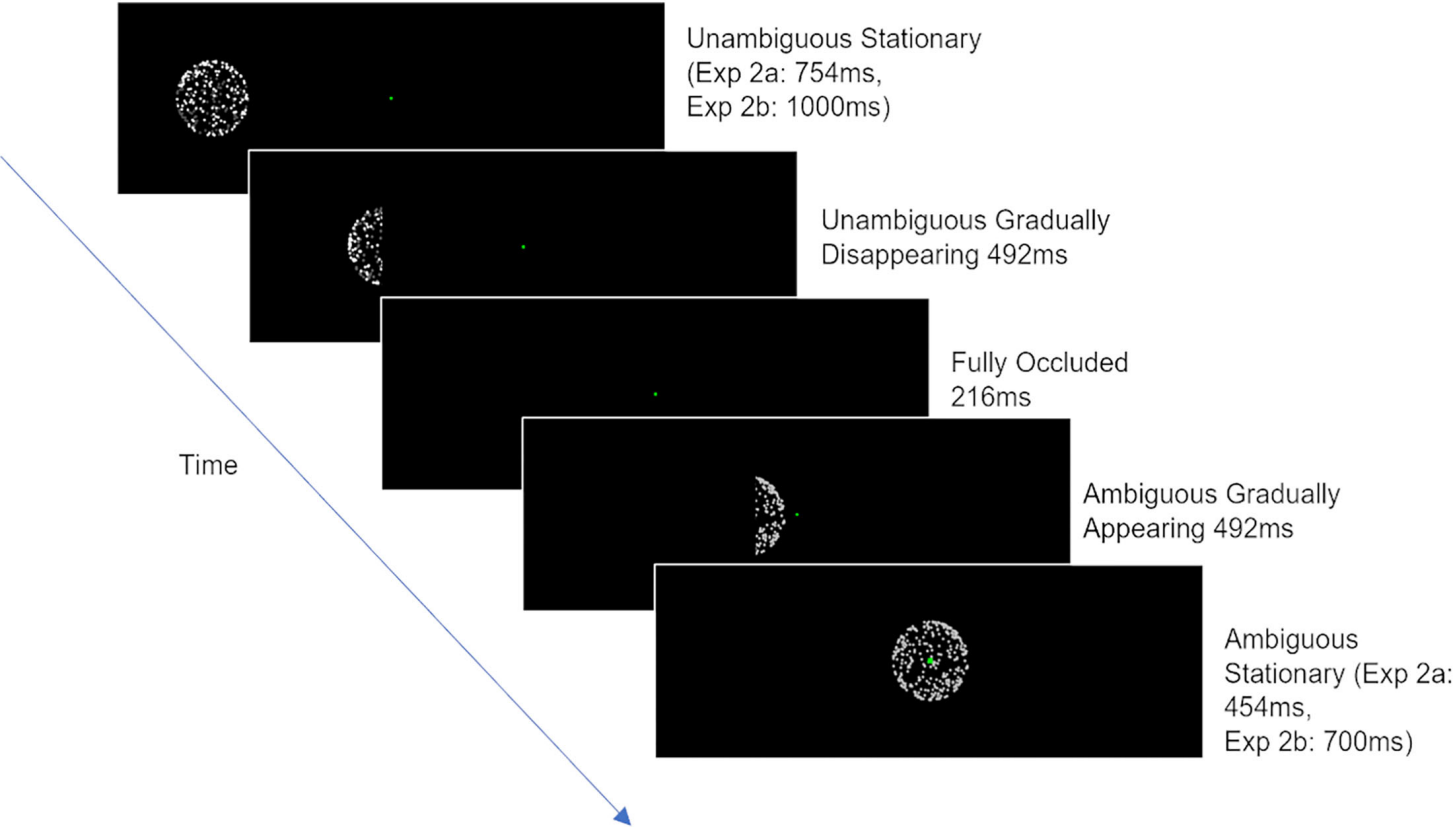
Example UA trial in Experiment 2 where translational motion was added to the sphere animations. In this instance, an unambiguous sphere was presented to the periphery and moved toward the central fixation point (note that, on other trials, the motion could be in the opposite direction, from the fixation point to the periphery). En route, the sphere was occluded for a short period before reappearing as an ambiguous stimulus moving to its final location.

In Experiment 1, the unambiguous prime and ambiguous probe spheres were presented to the same retinal location on each trial. To dissociate the influence of high-level mechanisms from lower level retinotopic factors, in this experiment we adapted the task such that the prime appeared at a different location from the probe, so local retinotopic effects of the prime could not influence perception of the probe. The unambiguous prime was shown at one location (fixation or periphery) and then moved to the other location (periphery or fixation, respectively) as it became ambiguous (see illustration in Figure 3). Under these conditions, any systematic effect must stem from a higher order (i.e., non-retinotopically organized) source.

In terms of precision-weighted predictive coding, presenting the unambiguous sphere to fixation should establish a precise prior. But, when the sphere moves to the periphery and becomes ambiguous, the sensory evidence it yields should be less precise than the prior. In this case, the precise prior should exert a strong influence on the construction of posterior estimates, so the ambiguous sphere is likely to be perceived as rotating in the same way as the prime (no switch).

Conversely, presenting the prime to peripheral vision creates a less precise prior (relative to a fixated prime). When the sphere moves to fixation and becomes ambiguous, the sensory evidence it yields is more precise than the prior. This leads to a posterior that, although still biased toward the primed direction, incorporates a comparatively higher probability of a switch in perception. (See Supplementary Section S1 and Supplementary Figure S2 for a more detailed explanation of this account.)

We therefore predicted that, if a high-level precision weighted mechanism can at least partially account for the effect observed in Experiment 1, then participants should be more likely to report switches on UA trials where the unambiguous prime is presented to peripheral vision (and then moves, becoming ambiguous at fixation), compared with when it is presented to fixation (and then moves, becoming ambiguous in the periphery). Methods and planned analyses for this experiment were preregistered on the Open Science Framework (https://osf.io/kc63w).

### Methods

#### Participants

Sixty-eight undergraduate psychology students and members of the community participated for course credit or shopping vouchers. Of this sample, 13 were excluded for catch trial performance, and one was excluded for failing to maintain fixation on more than 33% of trials. The final sample thus consisted of 54 participants (12 identified as male, 41 as female, and one as non-binary; mean age, 20.7 ± 3.70 years; by self-report, 49 were right handed, four were left handed, and one was ambidextrous). This sample was based on an estimated moderate effect size (*d* = 0.355) with 80% power (α = 0.05). We powered for a conservative effect size based on half the effect size observed in Experiment 1 (*d* = 0.71), given the likelihood that some of the original effect may be attributable to low-level retinotopic factors.

#### Stimuli, apparatus, and design

The test environment, stimuli, and design were the same as those used in Experiment 1, except for the following differences: The SFM sphere animations also contained a component of horizontal, translational motion such that the entire sphere moved at a constant speed of 4.17 dva/s either from the fixation point to the periphery or from the periphery to the fixation point.

In Experiment 1, stimuli were presented either at fixation or 5.0° to the left or right of fixation. In the present experiment it was important to avoid presenting the spheres to visual field regions between these points, in order to eliminate any effects of stimulation in the intervening retinal space. Therefore, while moving between the two locations, the sphere passed behind a rectangular occluder that had the same color and luminance as the background. This occluder was 3 dva wide, covering the full distance between the edge of a sphere at fixation and the inward edge of a sphere in the periphery. This meant that, for a short period on each trial, the sphere was completely hidden from view before emerging on the other side and continuing to its destination. Importantly, such brief occlusion maintains object constancy for moving objects (Scholl & Pylyshyn, 1999) (see also the Results section, below), which may be required for a top–down influence of object-level representations, as the sphere that emerges from behind the occluder may have to be perceived as the same one that initially disappeared behind it. We note that the occluder, which was not present in Experiment 1, introduces a new element that may, in itself, account for any difference between the effects observed in the two experiments. We addressed this issue explicitly in Experiment 3, where we replicated the location effect of Experiment 1 with occlusion similar to that used in Experiment 2.

#### Procedure

As in Experiment 1, all trials began with a 1300 ms fixation point. On each trial (except for the A trials), an unambiguously rotating sphere appeared for 1000 ms at its starting location (i.e., either the fixation point or 5 dva left or right of fixation). Next, the sphere moved horizontally towards its final location, gradually disappearing behind the occluding curtain over 492 ms, at which point it was fully occluded. After 216 ms of full occlusion (the duration it took the stimulus to travel to the other end of the occluder), the sphere emerged from the opposite side with either ambiguous (UA) or unambiguous rotation (UU and US) and moved horizontally for 492 ms until becoming fully visible at its final location. It remained at this final location for an additional 700 ms before disappearing (Figure 3). On the A trials, only the fixation point was visible until the ambiguous sphere emerged from behind the occluder, similar to the post-occluder phase of the other conditions. Note that the stimuli were stationary for the same durations as in Experiment 1, but trials here were longer overall due to the addition of motion periods. A 300 ms post-trial fixation period followed the offset of the probe sphere, and then the response screen appeared.

The number of trials and the distribution of conditions in this experiment were identical to those of Experiment 1. In this experiment, however, the peripheral location in each trial was blocked: Every trial within a given block either started or ended with the sphere animation in the same peripheral location (either left or right of fixation). This eliminated a difference in uncertainty between trials beginning at fixation (where the sphere could potentially move toward either direction) and trials beginning in the periphery (where the sphere would always move toward fixation). Blocking the peripheral location meant participants always knew which way a prime would move, thus removing a potentially confounding factor. Blocks were counterbalanced across participants by using either an AABBBBAA or a BBAAAABB block order, where A represents blocks using the left periphery and B represents blocks using the right. Participants completed eight blocks of 36 trials.

### Results

Trials and participants were excluded using the same criteria as Experiment 1, but participants were considered to have moved their eyes on trials where either the median or standard deviation of the horizontal deviation from the fixation point exceeded 1.35°. This criterion was more conservative than that used in Experiment 1 to account for the relatively greater number of eye-gaze samples collected on each trial, which increased as a function of the trial duration. Of the 288 trials collected from each participant, a mean of 16.1 trials were rejected from included participants’ data (range, 1–43).

As in Experiment 1, preliminary analyses indicated that it was appropriate to collapse across presentation side and rotation axis (see Supplementary Section S2).

**Figure 4.**
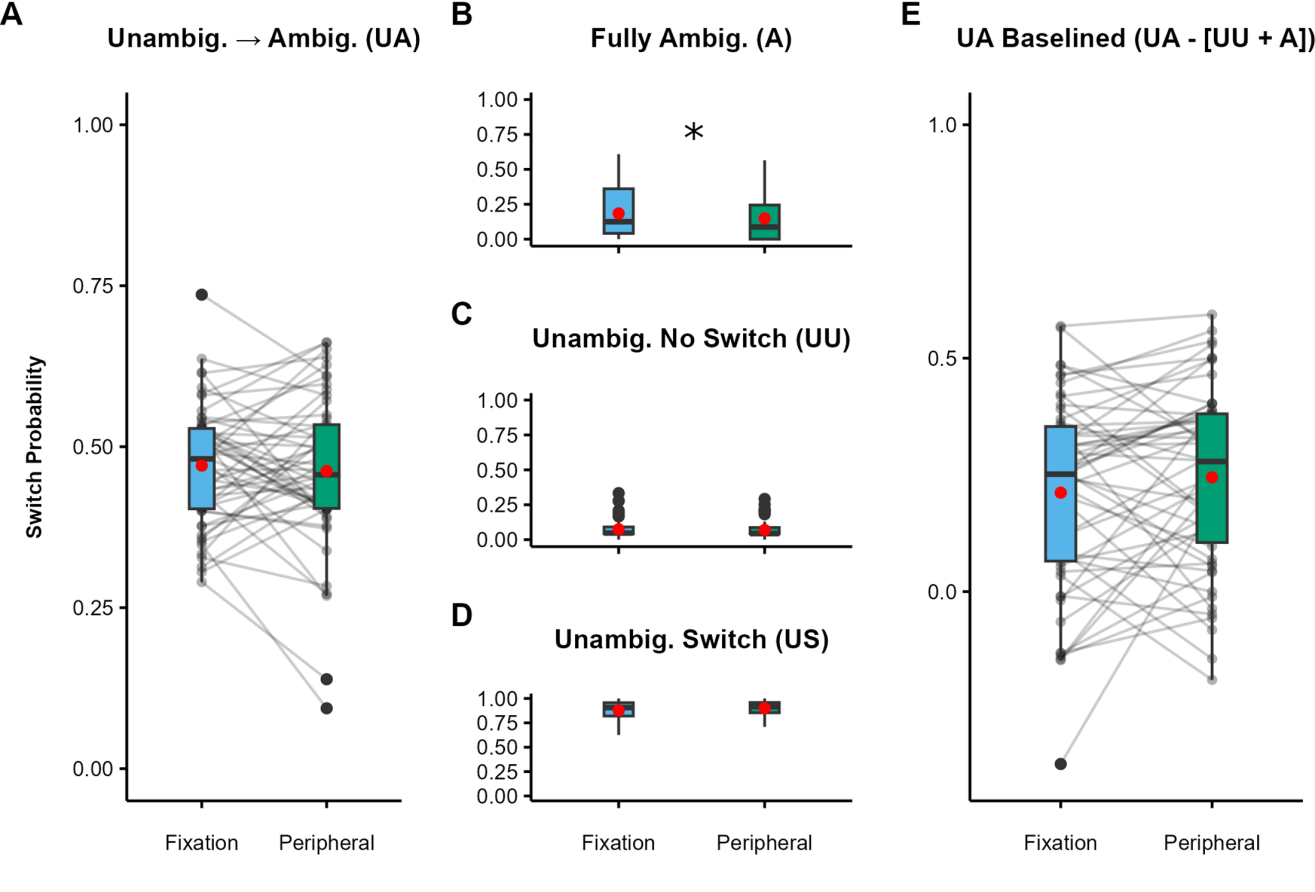
Experiment 2 results. (**A**–**D**) Switch probabilities for peripheral-to-fixation movement and fixation-to-peripheral movement in UA trials (**A**), A trials (**B**), UU trials (**C**), and US trials (**D**). (**E**) UA trials baselined by subtracting A and UU probabilities. The scale in panel E is extended to include negative values. Black dots indicate individual participants, red dots indicate condition means, boxes depict the IQR, and whiskers include all values < 1.5 × the IQR above or below the 75th or 25th percentile, respectively. *p < 0.05. Because the *x*-axis labels refer to the destination of the sphere animation (i.e., the location of the ambiguous sphere on UA trials), the hypothesized effect should show “fixation” bars as higher than “periphery” bars (opposite of the effect observed in Experiment 1).

For UA trials (Figure 4A), no significant difference in switch reports (*Z* = –0.66, *p* = 0.508) was observed between trials in which the sphere started in the periphery and moved to fixation (*M* = 0.47) and those in which it started at fixation and moved to the periphery (*M* = 0.46). We calculated Bayes factors to evaluate the strength of evidence for the null hypothesis. Bayes factors with values of under 1/3 are typically interpreted as good evidence for the null, values of over 3 are interpreted as good evidence for the alternative hypothesis, and intermediate values indicate that the evidence does not provide strong support for either hypothesis (Dienes, 2014). Our model for null hypotheses in this report was a standard Cauchy distribution centered on zero with a rate of 0.707. We used the raw effect on UA switch rates from Experiment 1 (0.14) as the prior; the Bayes factor (*BF*_10_ = 0.18) indicated support for the null hypothesis.

The probability of reporting a switch overall in the A condition was numerically higher than the same condition in Experiment 1; this can likely be attributed to the increase in overall stimulus duration. Switches were significantly more likely to be reported (*Z* = –2.80, *p* < 0.05) (Figure 4B) when the ambiguous sphere moved from behind the occluder into fixation (*M* = 0.19), compared with when it moved into the periphery (*M* = 0.13; *dz* = 0.21; 95% CI, 0.01–0.40).

As in Experiment 1, switches were rarely reported in the UU condition (Figure 4C). However, unlike Experiment 1, there was no significant difference in switch probabilities (*Z* = –0.46, *p* *= 0.*648) between the two movement conditions in this experiment (fixation to periphery, *M* = 0.07; periphery to fixation, *M* = 0.07).

Switches were reported in the vast majority of trials in the US condition (Figure 4D); as noted in Experiment 1, this was by design. A small difference between the two movement conditions was observed (*Z* = 1.8, *p* = 0.072) that approached, but did not reach statistical significance (fixation to periphery, *M* = 0.90; periphery to fixation, *M* = 0.88).

Finally, as in Experiment 1, we calculated a baselined measure of switch probabilities for each starting eccentricity in the UA condition by subtracting the corresponding switch probabilities observed in the UU and A conditions. As depicted in Figure 4E, the null effect of location persisted after baselining, with no significant differences, *t*(104) = 1.30, *p* = 0.197, between trials that started in the periphery (*M* = 0.21) compared with those that started at fixation (*M* = 0.24). Bayes factor analysis using the Experiment 1 raw difference between mean baselined UA switch rates at fixation versus periphery as the prior (0.09) indicated support for the null hypothesis (*BF*_10_ = 0.11).

Notably, the baselined UA switch rates confirm that the unambiguous spheres primed perception of the ambiguous spheres (and corroborate object constancy across locations). Switch rates were significantly below the 50% chance level (i.e., the ambiguous sphere was perceived as rotating in the same direction as the unambiguous prime) for trials that began in the periphery and ended at fixation (0.21), *t*(53) = 10.21, *p* < 0.001, *dz* = 1.39, 95% CI, 0.78–2.00), as well as for trials that began at fixation and ended in the periphery (0.24), *t*(53) = 9.70, *p* < 0.001, *dz* = 1.32, 95% CI, 0.71–1.92).

### Discussion

Under conditions that precluded the influence of low-level retinotopic factors, the results of this experiment do not corroborate the precision-weighted predictive coding account of the effect we observed in Experiment 1 (where switches following an unambiguous prime were more likely in peripheral compared with foveal vision). If the influence of high-level prior expectations on bistable awareness is precision weighted, participants should have reported more switches in UA trials when the unambiguous prime was presented in the periphery (where acuity and precision are lower) than when it was presented at fixation. However, no such difference in switch rates was observed.

Interestingly, baseline-corrected switch rates in the UA trials confirm the presence of a priming effect—ambiguous spheres were likely to be perceived as rotating in the same direction as the preceding unambiguous ones. The similarity between priming effects for peripheral and fixated primes, however, indicates that the priming is not influenced by the encoding precision of the prime and cannot account for the fixation versus periphery effect observed in Experiment 1. It is noteworthy that, in Experiment 1, switch rates were close to (and even exceeded) 50%, indicating more switching than priming. Because it is plausible that the same priming mechanism would have been engaged in that experiment, too, it is likely that the location effect in Experiment 1 was due to retinotopically specific factors that work in the opposite direction.

What factors are likely to yield this effect? One straightforward candidate is adaptation. There is already evidence that adaptation plays an important, although not exhaustive, role in the fluctuating perception of bistable imagery (Pearson & Brascamp, 2008). In Experiment 2, the results of the UU condition indicate that adaptation to non-retinotopic, object-level sphere representations did not differentially affect switch rates across the two horizontal movement conditions. Therefore, it seems plausible that lower level adaptation, perhaps to dominant motion cues (brighter dots) in the unambiguous priming sphere, may account for the fixation versus periphery effect of Experiment 1. If motion adaptation to our unambiguous stimuli was greater in the periphery, this would have led to larger peripheral aftereffects and thus to a stronger perceptual bias for opposite motion in ambiguous probes shown at the same location. In Experiment 3, therefore, we tested the hypothesis that our stimuli induced stronger motion aftereffects (MAEs) in the periphery than at fixation, and we investigated whether such differential adaptation could account for the visual field effect in Experiment 1.

Independently of MAEs, it is also possible that the occluder, which was not present in Experiment 1, was responsible for abolishing the location effect in Experiment 2. For example, the interaction between the edge of the occluder and the edge of the sphere stimulus may have disrupted the low-level encoding required for a stable SFM experience as it moved across the visual field. Experiment 3 addressed this possibility, as well.

## Experiment 3

The aims of Experiment 3 were twofold: First, we aimed to replicate the effect of location on switch rates observed in Experiment 1 while extending it to slightly different presentation conditions by testing whether it would occur in the presence of an occluder; if the effect did not replicate, then the occluder may explain the absence of a location effect in Experiment 2. Second, we aimed to clarify whether a retinotopically specific difference in the magnitude of motion adaptation induced by the unambiguous prime could explain the effect observed in Experiment 1. Previous work has demonstrated no difference in the magnitude of MAEs (an index of motion adaptation) between fixation and 5° eccentricity (Glasser, Tsui, Pack, & Tadin, 2011). Indeed, this was the reason we chose this eccentricity for our peripheral presentations; however, that work used different stimuli than ours, so the adaptation dynamics may also differ.

To address both aims simultaneously, we ran participants through two separate tasks:

1. The SFM task was a nearly direct replication of Experiment 2. However, this time the sphere stimuli remained stationery (as in Experiment 1), and the occluder moved over them. If the occluder was responsible for the absence of a location effect in Experiment 2, then there should be no effect of location in the UA condition of the present experiment. Conversely, if the occluder was not responsible, we expected to replicate the effect of location seen in Experiment 1.
2. The MAE task was designed to measure the magnitude of MAEs induced by the unambiguous sphere at each visual field location. We used the classic “nulling” paradigm (Blake & Hiris, 1993): On each trial, participants viewed the unambiguous SFM sphere, which acted as an adapter; following this, they saw a random dot stimulus (non-SFM) in which a proportion of the dots moved coherently and reported the perceived motion direction. Coherent motion could be in either the adapted or the opposite direction, enabling us to measure the point of subjective equality (PSE) between the two. The PSE corresponds to the strength of motion signal present in the random dot stimulus required to completely counteract the influence of a motion aftereffect on discrimination. If adaptation to the “front” surface of an unambiguous sphere causes stronger aftereffects in the periphery than at fixation, then participants’ PSEs should be a stronger motion signal in the adapted direction on peripheral compared with fixated trials, indicating a stronger MAE in the periphery (see Supplementary Section S3 and Supplementary Figure S8).

We expected to find more switches for peripheral than for fixated stimuli in the SFM task, replicating the effect observed in Experiment 1. If retinotopic motion adaptation differences accounted for the original effect observed in Experiment 1, we should also find stronger MAEs in the periphery than at fixation. Crucially, however, these two effects should also be positively correlated: The extent to which MAEs are stronger in the periphery than at fixation should predict the extent to which participants report more switches in the periphery than at fixation in the SFM task. Methods and planned analyses for this experiment were preregistered on the Open Science Framework (https://osf.io/wjv7h).

### Method

#### Participants

One hundred undergraduate psychology students and participants from the community voluntarily participated in both the SFM task and the MAE task, in exchange for course credit or supermarket vouchers. Three participants were excluded for moving their eyes too often in one or both tasks, 14 were excluded for catch trial failure in the SFM task, and two were excluded for poor performance in the MAE task (see Supplementary Section S3 for exclusion criteria), resulting in a final sample of 81 participants (24 identified as male, 55 as female, and two as non-binary; mean age, 21.64 ± 5.21 years; by self-report, 71 were right handed, nine were left handed, and one was ambidextrous). This sample size was determined via G*Power analysis as sufficient to detect a moderate correlation (*r =* 0.35) between task effects with 90% power (α = 0.05).

#### Stimuli, task design, and procedure

Participants completed both tasks over the course of a single 2-hour session. Each task took 40 to 50 minutes, and a short break was given between tasks. The order of tasks was counterbalanced among participants. Each task was preceded by a short practice, exposing participants to the various stimulus conditions in central and peripheral vision. As in Experiment 2, each task was divided into eight blocks of 36 trials.

#### SFM task

**Figure 5.**
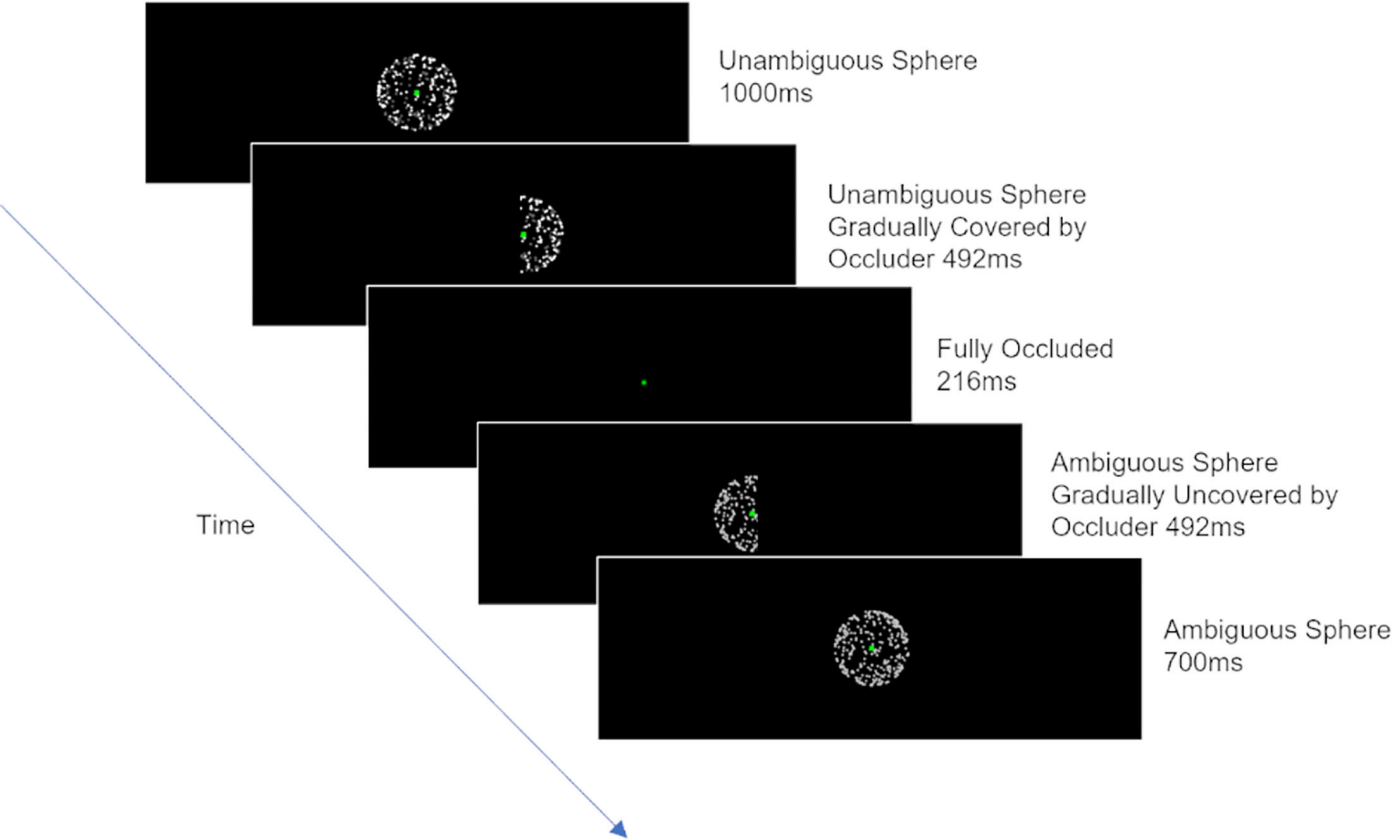
Example of a UA trial from the SFM task in Experiment 3 where we added translational motion to the occluder, instead of the sphere. In this instance, an unambiguous sphere is presented to fixation and is covered by a moving occluder. The sphere is fully occluded for a short period before being uncovered as an ambiguous sphere.

The test environment and stimuli were identical to those of Experiment 2, except for the position of the sphere, which remained fixed as the occluder moved horizontally across it (Figure 5). The motion of the occluder was designed to precisely emulate the interaction between the edge of the sphere and the occluder on each trial in Experiment 2 without changing the retinal location of the sphere animation. Accordingly, the translational speed of the occluder was equal to that of the sphere in Experiment 2 (4.17 dva/s), and the direction of motion relative to the sphere was identical to that of an equivalent trial in Experiment 2. For example, a UA trial that began at fixation before moving rightward in Experiment 2 can be considered as matched to a UA trial that remained at fixation while the occluder moved across it from right to left; the interactions between the rightward edge of the sphere and the leftward edge of the occluder were identical.

We used the same conditions as Experiments 1 and 2 and, as in Experiment 2, the side of the visual field in which the motion occurred was blocked to eliminate differential uncertainty about motion direction (this time of the occluder). Therefore, in half of the blocks, spheres appeared either to the left of fixation with the occluder approaching from the right or, alternatively, at fixation with the occluder approaching from the left. In the other half of the blocks, spheres appeared either to the right of fixation with the occluder approaching from the left or, alternatively, at fixation with the occluder approaching from the right. All other aspects of the task were identical to Experiment 2.

#### MAE task

We used unambiguously rotating SFM spheres as adapter stimuli. These adapters were identical to the unambiguous spheres used in Experiments 1 and 2 and the SFM task of the present experiment. Following adaptation on each trial, participants viewed motion discrimination probes—random-dot kinematograms (RDKs), similar to the classic dot-motion stimuli used to study motion processing (Blake & Hiris, 1993; Britten, Shadlen, Newsome, & Movshon, 1992; Castet, Keeble, & Verstraten, 2002). These RDK stimuli contained 150 gray dots that were identical to those used in the ambiguous SFM sphere in Experiments 1 and 2 and the SFM task of the present experiment. These dots were randomly positioned within a circular aperture (radius, 1.0 dva) whose outer edge was occluded, leaving an observable 0.87-dva radius aperture. A coherent motion signal was added to each RDK probe by randomly selecting a fixed percentage of dots to move in the signal direction between each pair of consecutive frames while the remaining dots moved in random directions. Importantly, this meant that a new set of coherent-motion dots was selected with every frame transition, so participants could not generally discriminate motion direction by following the movement of a single dot. The positions of the dots were updated in each consecutive frame, such that they moved with a speed of 0.73 dva/s. Dots that moved beyond 1.0 dva from the center of the stimulus were repositioned using a “wraparound” procedure (see Supplementary Section S3 and Supplementary Figure S9 for full stimulus details).

Nulling paradigms typically require participants to observe adapter stimuli for several seconds on each trial, but we wished to ensure that MAEs were of equal strength to those that may have been induced in the SFM task. Therefore, trials in the MAE task were designed to be as similar to those of the SFM task as possible, including the use of a horizontally moving occluder. Unambiguous sphere adapters always rotated on one of the four non-cardinal axes used in the previous experiments. Signal dots in the RDK probes always moved in either the same or reverse direction as the bright dots (i.e., the “front” face) of the unambiguous adapter.

Trials were identical to UA trials in the SFM task except for the use of RDKs instead of the ambiguous sphere probes. After each trial, rather than reporting switches, participants were instructed to report the direction of motion they perceived in the probe. The response prompt contained two arrows, representing each of the two perceivable motion directions. These prompts were designed such that participants always reported leftward motion (left–up or left–down) using their left hand (the [A] key) and rightward motion (right–up or right–down) using their right hand (the [L] key).

Unlike the SFM task, each participant observed SFM adapters in only one rotation direction to minimize interference between different adaptation directions, with the four possible directions counterbalanced across participants. Of the included sample, 20 participants viewed adapters whose “front” face moved at an angle of 45°, 22 viewed adapters at 135°, 18 viewed adapters at 225°, and 21 viewed adapters at 315°. For the RDK probe, we used six levels of coherent motion signal (0.1, 0.2, 0.3, 0.4, 0.5, and 0.9) for each of the two directions. We denoted positive signal values as motion in the same direction as the adapter and negative values as the opposite direction. The first five values for each direction (± 0.1, 0.2, 0.3, 0.4, and 0.5) were selected because they covered the dynamic range (i.e., the range of motion signal values that spans chance to perfect performance) for the experimenters and pilot participants. The extreme values (±0.9) were included as catch trials where nearly perfect performance was expected.

Half of all trials were presented to the fixation point, and the remaining half were presented 5.0 dva into the left or right periphery. The side of the visual field in which peripheral trials occurred was blocked (AABBBBAA or BBAAAABB) to eliminate confounding differences in uncertainty about the motion of the occluder and to eliminate differences in cumulative, intertrial adaptation between fixated and peripheral locations over the course of each block. Each participant completed 288 trials (12 motion signal levels × 2 levels of stimulus location × 12 repetitions).

### Results

#### SFM task

**Figure 6.**
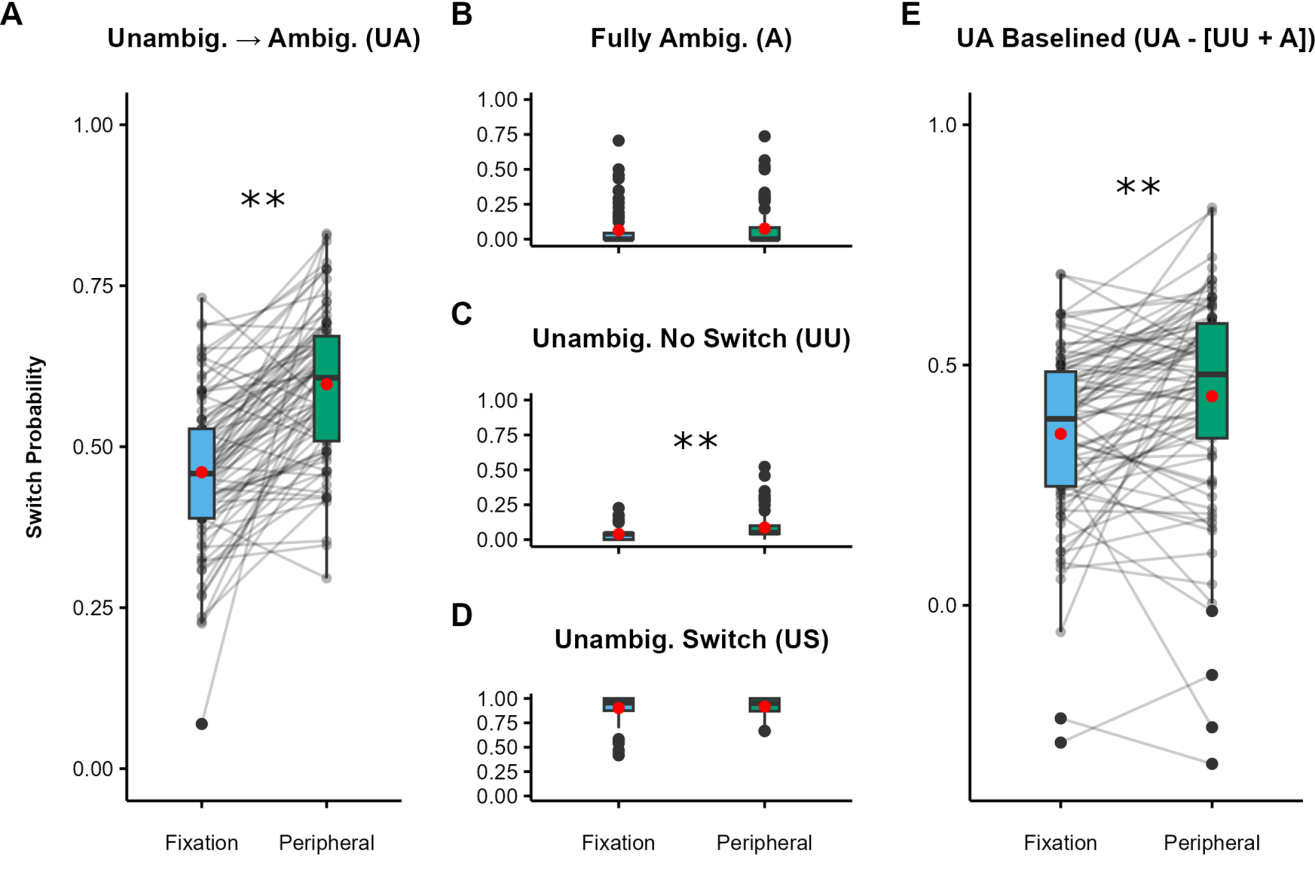
SFM task results for Experiment 3. (**A**–**D**) Switch probabilities for fixated and peripheral trialsUA condition (**A**), A condition (**B**), UU condition (**C**), and US condition (**D**). (**E**) UA trials baselined by subtracting the A and UU switch probabilities. The scale in panel E is extended to include negative values. Black dots indicate individual participants, red dots indicate condition means, boxes depict the IQR, and whiskers include all values < 1.5 × the IQR above or below the 75th or 25th percentile, respectively. **p < 0.001.

Preliminary analyses indicated that it was appropriate to collapse across presentation side and rotation axis (see Supplementary Section S2). Critically, the effects of location replicated those observed in Experiment 1. In the UA condition (Figure 6A), participants were significantly more likely (*Z* = 14.42, *p* < 0.001) to report a switch in sphere rotation direction when stimuli were presented to the periphery (*M* = 0.60) compared with fixation (*M* = 0.46; *dz* = 1.03; 95% CI, 0.81–1.41).

In the A condition, switch report rates were low and did not differ for peripheral (*M* = 0.08) and fixated (*M* = 0.06) stimuli (*Z* = 1.67, *p* = 0.95) (Figure 6B). Switch rates in the UU condition (Figure 6C) were also low; importantly, however, as in Experiment 1, participants were significantly more likely (*Z* = 5.95, *p* < 0.001) to report switches on peripheral UU trials (*M* = 0.09) compared with fixated UU trials (*M* = 0.04; *dz* = 0.49; 95% CI, 0.32–0.66), indicating possible differences between locations in adaptation to the unambiguous sphere.

Finally, by design as in the previous experiments, the overall switch probability for the US condition (Figure 6D) was very high, with no significant difference in switch probability (*Z* = 1.67, *p* = 0.096) between peripheral (*M* = 0.92) and fixated (*M* = 0.90) trials.

We again calculated baseline-corrected switch rates for the UA condition (Figure 6E). The effect of location was preserved, with significantly higher switch rates for peripheral trials (*M* = 0.44) compared with fixated trials (*M* = 0.36), *t*(158) = 3.81, *p* < 0.001, *dz* = 0.42, 95% CI, 0.20–0.66.

#### MAE task

To assess MAEs, we used the point of subjective equality (PSE), the strength of motion signal present in the RDK probe for which observers report each possible probe direction with equal probability after adaptation to one of the directions. Positive PSEs indicate that equality occurs when probe motion is in the same direction as the adapter, indicating motion adaptation; more strongly positive PSEs indicate greater motion adaptation. A negative PSE would indicate that equality occurs when probe motion is in the opposite direction to the adapter, an unlikely occurrence following adaptation. Two PSEs (one for the periphery and one for fixation) were calculated for each participant by fitting logistic functions to responses to the different motion coherence levels. Peripheral PSE calculations collapsed across the left and right visual field (a preliminary paired *t*-test revealed no significant difference in PSEs between the two peripheral locations), *t*(80) = 1.27, *p* *= 0.*206. Logistic functions were fit using the *quickpsy* package in R (R Foundation for Statistical Computing, Vienna, Austria) with default guess and lapse rates of 0 (Linares & López-Moliner, 2016).

Crucially, the PSE for peripheral stimuli in the MAE task (*M* = 13% motion coherence) was significantly higher than the PSE for stimuli presented at fixation (*M* = 7%), *t*(158) = 5.30, *p* < 0.001, *dz* = 0.51, 95% CI, 0.36–0.86. This confirms that motion adaptation to our unambiguous sphere stimuli was indeed greater in the periphery than at fixation.

**Figure 7.**
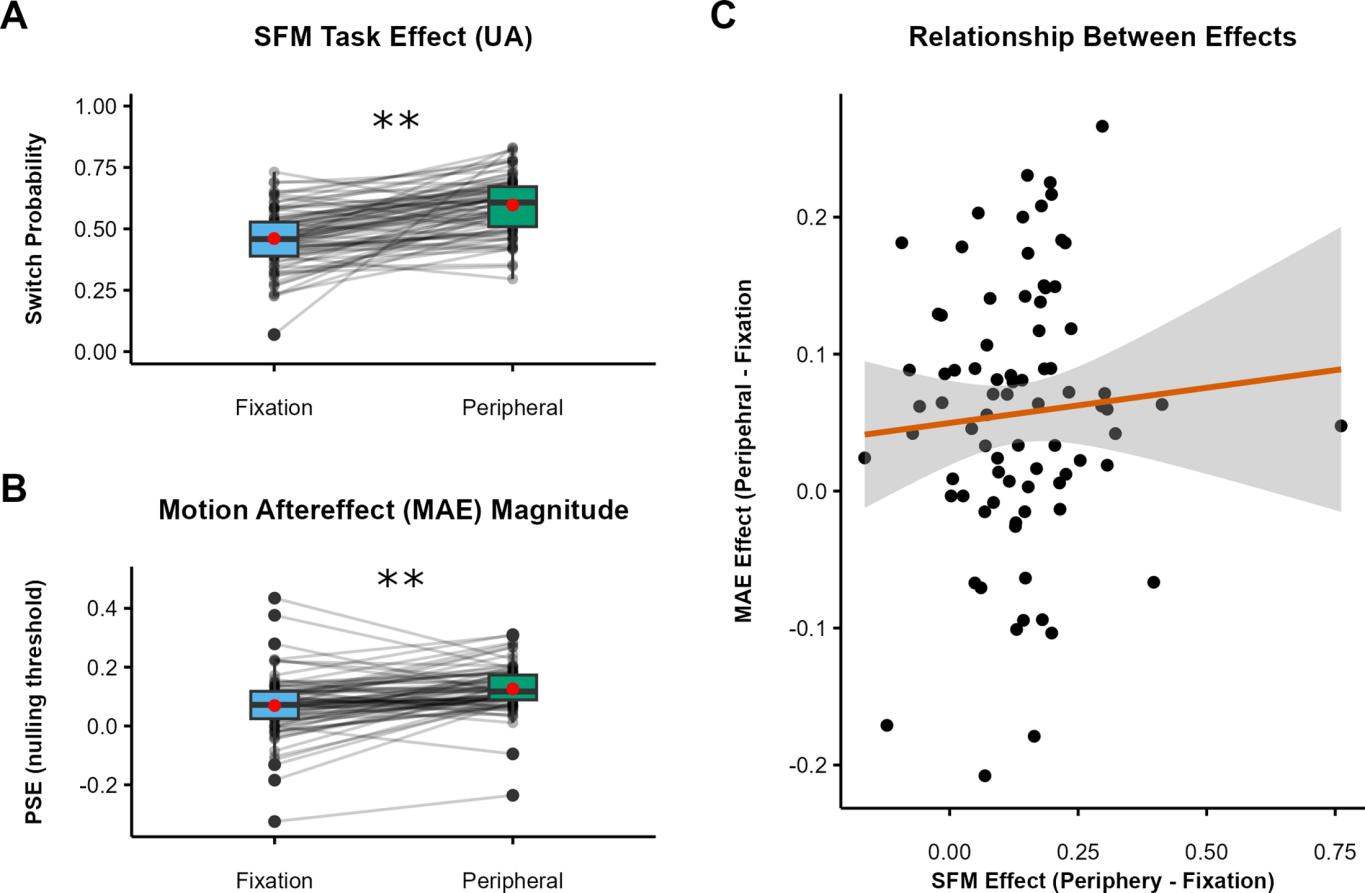
Relationship between effects for Experiment 3. (**A**) The effect of eccentricity on switch probability for UA trials in the SFM task (repeated, for clarity, from Figure 6A). (**B**) The effect of eccentricity on observers’ PSE, indicating stronger MAEs in peripheral vision (note that the *y*-axis scale is different from panel **A**). Black dots indicate individual participants, red dots indicate condition means, boxes depict the IQR, and whiskers include all values < 1.5 × the IQR above or below the 75th or 25th percentile, respectively. (**C**) The relationship between the two effects. Each data point reflects one participant. The gray region indicates the 95% CI around linear model estimates. **p < 0.001.

The hypothesis that motion adaptation may underlie the periphery versus fixation effect of the SFM task predicts not only the location effect observed in each task but also a positive correlation between the magnitudes of the two effects. Next, we examined this correlation. For illustration, Figure 7A shows the mean switch rate for the UA condition in the SFM task (repeated from Figure 6A), Figure 7B shows the mean PSEs for the MAE task, and Figure 7C shows the correlation between the two effects. The SFM effect was calculated for each participant as the difference in switch probability between peripheral and fixated UA trials, and the MAE effect was calculated for each participant as the difference in PSEs between peripheral and fixated trials. To our surprise, although the effect of each task was in the expected direction, the two effects were not correlated, *r*(79) = 0.07, *p* = 0*.*266. We also examined each location separately and did not find any correlation between switch probabilities on peripheral UA trials and peripheral PSEs, *r*(79) = 0.05, *p* = 0*.*639, nor between switch probabilities on fixated UA trials and PSEs at fixation, *r*(79) = 0.2, *p* = 0.072.

As an exploratory analysis, we asked whether the magnitude of participants’ MAEs at each visual-field location could explain any of the variability in raw responses on UA trial tasks. We fit a logistic mixed model to the trial-level responses in the SFM task, including eccentricity, the PSE from the MAE task (computed independently for fixated and peripheral trials), and their interaction as fixed effects (formula: SFM response ∼ eccentricity × PSE) and participant number as a random effect (formula: ∼1|ID). Full model outputs are available in Supplementary Section S5. This analysis revealed a small, near-significant main effect of PSE on responses in the SFM task such that a positive shift of 1 *SD* in the PSE (*SD* = 0.10) was associated with a 7% increase in the odds of reporting a switch (standard odds ratio = 1.07; *p* = 0.052). There was no interaction between PSE and eccentricity (*p* = 0.301). These results indicate that a larger motion adaption effect was associated with a slightly elevated chance of reporting switches in general, but there is no evidence this contributed to a systematic difference across eccentricities. A similar analysis—this time using the change in PSE between peripheral and fixated trials as a fixed effect (formula: SFM response ∼ eccentricity × ΔPSE + 1|ID)—revealed no significant main effect (*p* = 0.146) or interaction (*p* = 0.359).

### Discussion

Experiment 3 successfully replicated the effect of eccentricity on switch probability found in Experiment 1: Participants were more likely to report switches in sphere rotation when an ambiguous probe followed an unambiguous prime in the periphery than at fixation. The effect was found in the presence of a moving occluder, ruling out the possibility that the occluder itself prevented location-based effects in Experiment 2.

We hypothesized that stronger adaptation to motion signals in the periphery might explain why switches are reported more frequently in this location. Stronger adaptation in the periphery to the motion of the high contrast “front” surface of the unambiguously rotating sphere would create a stronger perceptual bias toward opposite motion when viewing the ambiguous probe. We tested motion adaptation to our unambiguous spheres in a separate MAE task and indeed found stronger MAEs in the periphery than at fixation. However, if differences in MAEs were responsible for the effects of location on SFM switching, we would expect individuals with large MAE differences to also exhibit large differences in SFM switch probabilities, and vice versa. We found no such association between the two effects, suggesting that each may arise via a separate mechanism.

## Experiment 4

Although Experiment 3 showed that MAEs to our unambiguous stimuli were greater in the periphery than at fixation, it also showed that this did not account for the periphery versus fixation effect on SFM switching. In Experiment 4, we therefore turned to another plausible factor that may account for this effect: fixation stability, the magnitude and frequency of involuntary fixational eye movements, such as microsaccades and drifts, that occur while a participant maintains fixation. In the preceding experiments, participants were instructed to fixate on a central point and we excluded trials where participants made overt saccades away from this point. However, it is possible that, in the included trials, smaller fixational eye movements were systematically different for fixated versus peripherally presented stimuli. Specifically, it may be more difficult to maintain fixation when task-relevant stimuli are presented in the periphery than at fixation; this may have led to more and larger fixational eye movements during peripheral stimulus presentations. This may result in less stable stimulus encoding, which in turn might drive the elevated switch rate. In support of this idea, previous research has identified lower fixation stability for covertly attended peripheral stimuli (Raveendran, Krishnan, & Thompson, 2020). Moreover, other researchers have demonstrated effects of blinks, saccades, and microsaccades on bistable motion perception (Baker & Graf, 2010; Brych, Murali, & Händel, 2021).

Unfortunately, in the previous experiments, eye-movement data were used to detect breaks from fixation but were not retained. We therefore ran Experiment 4, in which we replicated Experiment 1 and collected detailed eye-position measurements in order to determine whether a decrease in fixation stability during peripheral stimulus presentations might explain the increase in switch rates. To measure fixation stability, we used the bivariate contour ellipse area (BCEA) of gaze coordinates for each trial (Raveendran et al., 2020), a measure that captures the distribution of eye movements over a defined period. Computation of this measure is detailed in Equation 1, where σ*_x_* and σ*_y_* correspond to standard deviations of the horizontal and vertical gaze positions, respectively; ρ corresponds to the Pearson correlation coefficient between horizontal and vertical gaze positions; and χ^2^ is the chi-square value (2 degrees of freedom) corresponding to 1 *SD* (i.e., a probability of 0.682). Therefore, the BCEA is the area of the ellipse that encompasses 68% of gaze positions within a trial; higher BCEAs correspond to a less stable fixation pattern.

(1) $$\begin{array}{c} \mathrm{BCEA}=\pi \chi^{2}\sigma_{x}\sigma_{y}\sqrt{(1-\rho^{2}})\; \end{array}$$

If a systematic difference in fixation stability between peripheral and fixated trials was responsible for the periphery versus fixation effect on switch probability, then BCEAs in the periphery should be larger than at fixation, and BCEA differences between the two locations (peripheral – fixated) should correlate positively with the effect of location on UA trials. Methods and planned analyses for this experiment were preregistered on the Open Science Framework (https://osf.io/crdm2).

### Method

One hundred and thirty undergraduate and community participants completed the task. Forty-one were excluded from analysis for catch trial failure, and eight were excluded for technical failures during data collection, resulting in a final sample size of 81 (20 identified as male, 60 as female, and one as non-binary; mean age, 20.07 ± 3.83; by self-report, 73 were right handed, five were left handed, and three were ambidextrous). Methods were identical to those in Experiment 1, with the following exceptions: Participants were instructed to avoid blinking during stimulus presentation and to try to only blink during response periods or between trials (trials in which the participant blinked were excluded, per our preregistered exclusion criteria). Binocular eye-gaze coordinates were recorded (sampling rate, 500 Hz) using a desktop mounted EyeLink 1000 eye tracker.

### Results

This time, preliminary analyses revealed that switch reports varied for different rotation axes in the UA condition; however, there was no significant interaction between rotation axis and visual field location. The effect of location on switch reports was consistent across different rotation axes (for full details, see Supplementary Section S2 and Supplementary Figures S6 and S7). We also found a difference between switch probabilities in peripheral UA trials presented to the left and right hemifields; however, the periphery fixation pattern of switch reports was present for both sides, and further analyses revealed that effect sizes were comparable, although smaller, for right versus fixation than left versus fixation (see Supplementary Section S2). As the effects of rotation axis and presentation side do not directly impact the hypotheses we tested, their full details are reported in the Supplementary Materials; for consistency with the previous experiments, below we report results collapsed across left and right peripheral locations and rotation axis.

**Figure 8.**
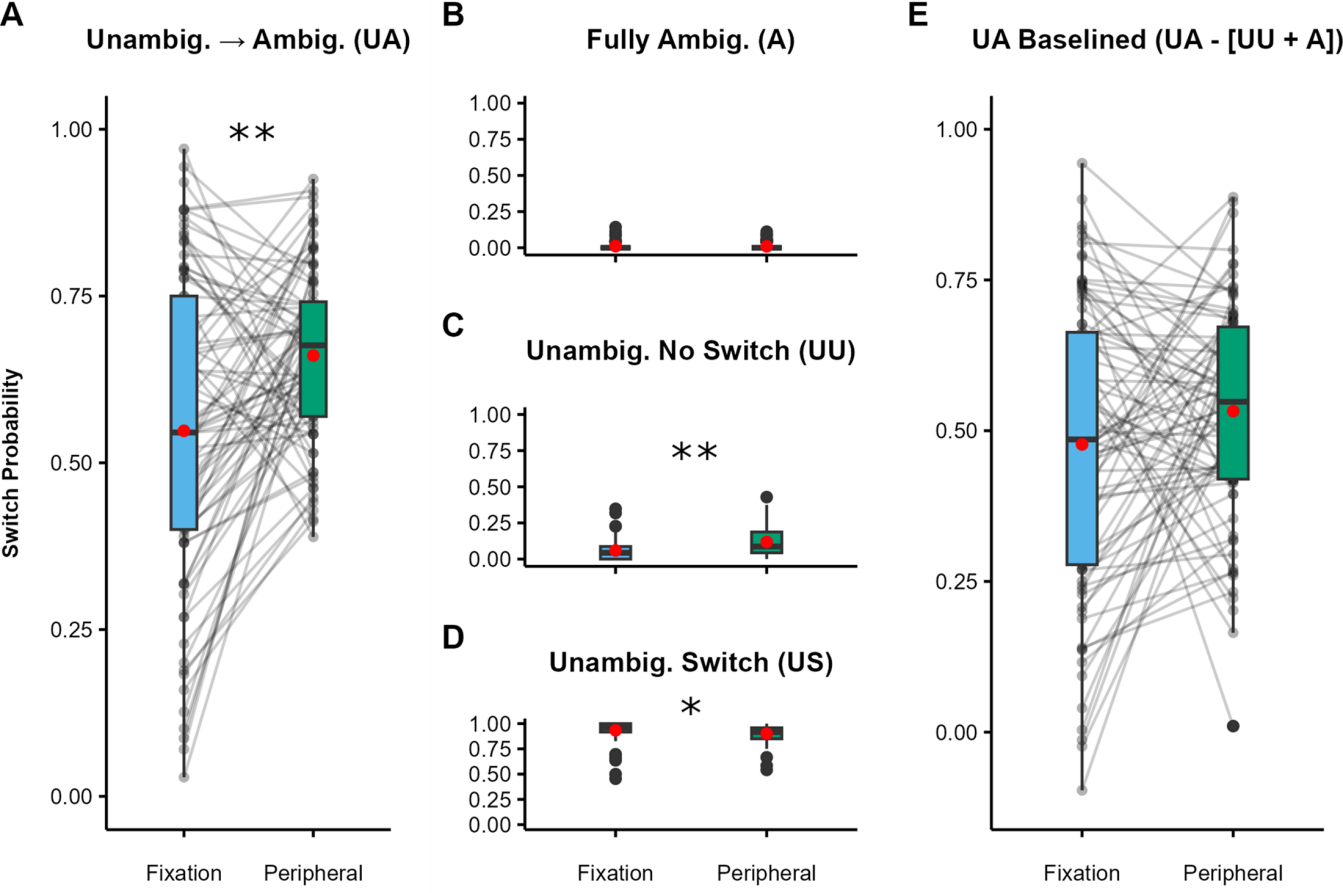
SFM task results for Experiment 4. (**A**–**D**) Switch probabilities for fixated and peripheral trials: UA condition (**A**), A condition (**B**), UU condition (**C**), and US condition (**D**). (**E**) UA trials baselined by subtracting the A and UU switch probabilities. Note the adjusted scale in panel E to accommodate negative values. Black dots indicate individual participants, red dots indicate condition means, boxes depict the IQR, and whiskers include all values < 1.5 × the IQR above or below the 75th or 25th percentile, respectively. **p < 0.001, *p < 0.05.

Replicating Experiments 1 and 3, participants were significantly more likely (*Z* = 12.73, *p* < 0.001) to report a switch in rotation for UA trials presented to the periphery (*M* = 0.66) compared with fixation (*M* = 0.54) (Figure 8A), although the effect size was somewhat smaller in this experiment than in our previous findings (*dz* = 0.45; 95% CI, 0.25–0.67).

In the A condition (Figure 8B), participants were again equally likely (*Z* = –0.51, *p* = 0*.*612) to report spontaneous switches on peripheral trials (*M* = 0.01) compared with fixation (*M* = 0.01). As in Experiments 1 and 3, on UU trials (Figure 8C), participants were more likely (*Z* = 6.49, *p* < 0.001) to report switches on peripheral (*M* = 0.12) compared with fixated (*M* = 0.06) trials, indicating possible differences in adaptation to the unambiguous sphere (*dz* = 0.80; 95% CI, 0.32–0.66). For US trials (Figure 8D), the overall switch probability was again very high by design. There was a small yet significant difference (*Z* = –3.84, *p* < 0.001) in switch probability such that switches were less likely on peripheral trials (*M* = 0.90) compared with fixated trials (*M* = 0.93; *dz* = 0.24; 95% CI, 0.02–0.54).

Importantly, consistent with Experiments 1 and 3, the location effect persisted when using our baselined measure (Figure 8E) to compare peripheral trials (*M* = 0.53) to fixated trials (*M* = 0.48), *t*(158) = 1.72, *p* = 0.087, *dz* = 0.19, 95% CI, 0.03–0.43, although it fell short of statistical significance.

**Figure 9.**
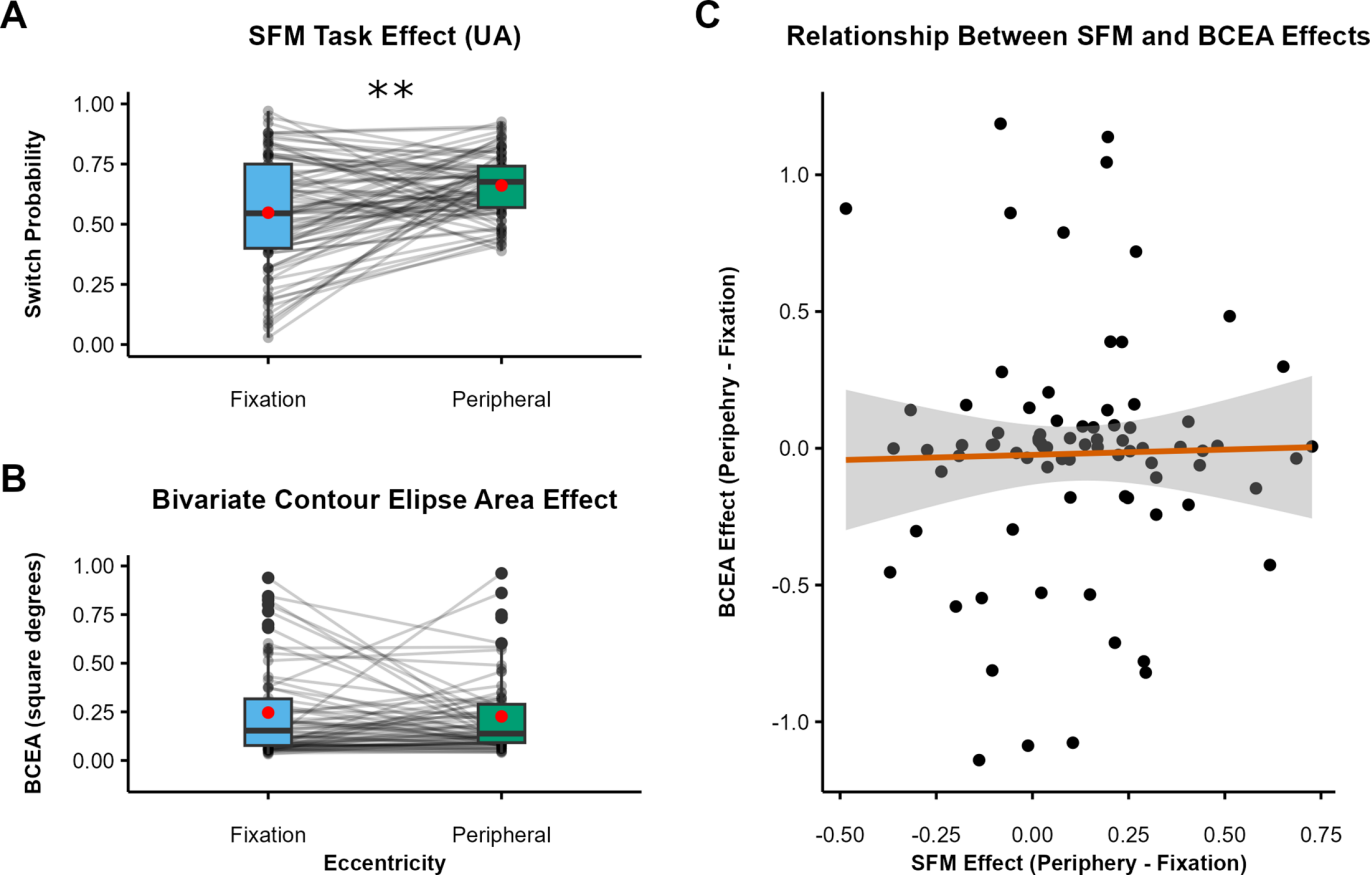
Relationship between effects for Experiment 4. (**A**) The effect of eccentricity on switch probability for UA trials in the SFM task (repeated, for clarity, from Figure 8A). (**B**) The effect of eccentricity on observers’ BCEA. Black dots indicate individual participants, red dots indicate condition means, boxes depict the IQR, and whiskers include all values < 1.5 × the IQR above or below the 75th or 25th percentile, respectively. (**C**) The relationship between the two effects. Each data point reflects one participant. The gray region indicates the 95% CI around linear model estimates. **p < 0.001.

Next, we examined whether the BCEA measure of fixation stability could account for the SFM switching effect of location. For illustration, Figure 9A shows the mean switch rate for the UA condition in the SFM task (repeated from Figure 8A). Figure 9B shows the mean BCEAs at the two locations; contrary to our predictions, there was no significant difference in the BCEA for fixated (*M* = 0.33 deg^2^) versus peripheral (*M* = 0.35 deg^2^) UA trials, indicating that fixation stability was equivalent at the two locations, *t*(158) = 0.39, *p* = 0.697. Nonetheless, we examined the correlation between the two measures. Even in the absence of mean BCEA differences, a positive correlation between BCEA and SFM switching location effects would suggest a common mechanism. However, there was also no correlation between differences in BCEA and differences in switch rate between fixated and peripheral stimuli, *r*(79) = 0.02, *p* = 0*.*879) (Figure 9C).

As an exploratory analysis, we asked whether the magnitude of participants’ BCEAs at each visual-field location could explain any of the variability in raw responses on UA trial tasks. We fit a logistic mixed model to the trial-level responses in the SFM task, including eccentricity, the BCEA (computed at the trial level), and their interaction as fixed effects (formula: SFM response ∼ eccentricity × PSE) and participant number as a random effect (formula: ∼1|ID). Full model outputs are available in Supplementary Section S5. Results indicated no effect of BCEA on responses (*Z* = 0.78, *p* *= 0.*437) and no interaction with stimulus eccentricity (*Z* = –0.81, *p* = 0.426).

A modulatory influence of fixation stability on the effect of location may be apparent if we separate the trials into the different perceptual outcomes: If fixation stability affects the probability of a switch—and, specifically, if switches are more likely when fixation is less stable—then there may be larger BCEA values in trials in which a switch occurred compared with those in which it did not. Furthermore, BCEA differences between switch and no-switch trials would be larger in the periphery than at fixation, explaining the effect of location on switch rates. To address this possibility, we entered BCEA measurements on UA trials into a repeated-measures ANOVA with the factors of stimulus eccentricity (fixation vs. periphery) and participant report (switch vs. no-switch). Although there was a significant interaction between stimulus eccentricity and participant response, *F*(1, 80) *=* 4.15, *p* < 0.05, η^2^ = 0.05 (see Supplementary Figure S10), no pairwise differences were statistically significant (all *p* > 0.05, Tukey-corrected for multiple comparisons); notably, contrary to our prediction, BCEA values in the periphery were numerically larger for no switch trials than switch trials. Fixation stability is thus unlikely to explain the location effect.

### Discussion

Experiment 4 provides a third replication of the effect of eccentricity on switch probability. We predicted that, if less stable fixation patterns in peripheral vision were responsible for the effect on switch rates, then the difference in BCEA between peripheral and fixated trials should predict the corresponding difference in switch rates. Instead, we found no overall difference in BCEA, nor were participants who had a larger difference more likely to have a greater difference in switch probability. The effect of location on switch rates is thus unlikely to be attributable to differences in eye movements.

The absence of BCEA differences between fixation and periphery contrasts with work by Raveendran et al. (2020), who reported less stable fixation patterns for stimuli presented at 5° eccentricity. Importantly, however, their measure of fixation stability was calculated over multiple trials comprising 15 seconds of gaze data, whereas our calculations were for individual trials lasting only 1.7 seconds. It is conceivable, therefore, that observable spatial differences in fixational stability only emerge over extended periods of fixation.

## General discussion

Differences between foveal and peripheral vision may have various influences on the contents of our conscious experiences. In the four experiments reported above, we examined one such influence, investigating how prior information may affect awareness differently at fixation versus the periphery. To do so, we measured, at each location, the influence of unambiguous SFM primes on the conscious perception of subsequent ambiguous stimuli. Experiment 1 found that observers’ awareness of the motion of ambiguous spheres was more likely to be biased away from an unambiguous prime sphere when stimuli were presented to peripheral vision than to fixation; this location effect was firmly established by replications in Experiments 3 and 4. We tested three possible mechanisms that may underlie the location effect: Experiment 2 demonstrated that high-level (non-retinotopic) object representations allowed for priming across locations but ruled out precision weighting of such representations as the underlying mechanism of the location effect of Experiment 1. Experiment 3 demonstrated a fixation-periphery difference in motion adaptation, but ruled out this differential motion adaptation as the underlying mechanism of the location effect. And, finally, Experiment 4 showed that the location effect is not attributable to differences in fixation stability for foveal compared with peripheral stimuli.

The three independent demonstrations of the location effect (in Experiments 1, 3, and 4) indicate that it is robust, but its underlying mechanism remains elusive. The potential mechanisms we tested and ruled out were plausible in light of theoretical considerations and previous empirical work and range from high-level, non-retinotopic predictive influences (of precision-weighted representations, Experiment 2), through feature-specific retinotopic adaptation (to motion, Experiment 3), to low-level quality of the visual signal (fixation stability, Experiment 4). Although many other mechanisms could be addressed in further work, at this point it should be acknowledged that the effect may reflect fundamental differences in characteristics of the visual processing infrastructure (e.g., the differential dominance of magno- and parvocellular processing streams) dedicated to different locations in the visual field.

In Experiment 2, priming was evident when the stimulus moved between the unambiguous-prime and ambiguous-probe phases, consistent with previous research demonstrating that predictive context facilitates perceptual content (Brascamp et al., 2018; Denison et al., 2011; Pearson & Brascamp, 2008; Weilnhammer et al., 2017; Weilnhammer et al., 2021; but see Pastukhov, Prasch, & Carbon, 2018, who found that complete occlusion of moving stimuli abolished persistence of bistable rotation direction). However, the priming was similar in magnitude regardless of whether the prime appeared at fixation or in the periphery, suggesting that although top–down priors appeared to play a general role, the location at which the priors were encoded (and thus their precision) did not affect their weight in constructing awareness. These results are consistent with the possibility that the priming effect may be impacted solely by the conscious interpretation of the prime (Weilnhammer, Stuke, Sterzer, & Schmack, 2018) rather than its precision. Conversely, they conflict with the idea that perceptual content can be modeled by a Bayesian posterior calculated as the precision-weighted average of priors and sensory input (Hohwy et al., 2008).

How can this be reconciled with previous research that has successfully described the dynamics of bistable SFM using Bayesian prediction-error modeling (Schmack, Weilnhammer, Heinzle, Stephan, & Sterzer, 2016; Weilnhammer et al., 2017; Weilnhammer et al., 2021)? Notably, the focus of these previous studies was on the dynamics of perceptual reporting across extended periods of stimulus exposure (up to several minutes at a time), modeling cumulative perceptual statistics rather than measuring the specific effects of immediate prior stimulation. Considering our results, it may be the case that the visual system applies precision weighting to cumulative perceptual history acquired over broader temporal scales, but not to individual instances of transient visual priming. Research in this domain typically treats the precision of priors as dynamic model parameters that diminish over time as a function of the amount of residual evidence available for the opposite percept (e.g., Weilnhammer et al., 2017). By contrast, our manipulation of visual field location directly affected the veridical precision of the visual information used to encode the prior. That our findings do not align with others may thus not be very surprising, given these different treatments of the prior precision construct. Visual acuity at different locations and statistical regularities in perceptual history are substantially different operationalizations of precision, and questions of how and at what level precision is represented in the brain, as well as when and to what extent it is reflective of the veridical precision of sensory information, are all the topic of ongoing discussion in the literature (Yon & Frith, 2021).

Previous work has noted two mechanisms that might contribute to priming effects of history on percept selection: a quickly decaying effect of neural persistence whereby the previously experienced stimulus attracts immediate selection of a new stimulus, and a weaker, slowly decaying attractive effect of perceptual memory (Pastukhov & Braun, 2013; Pearson & Brascamp, 2008). The current study investigated what happens when an unambiguous stimulus changes into an ambiguous one, either instantly or following brief occlusion, and thus separated prime and probe stimuli for a maximum of 216 ms (Experiments 2 and 3). Therefore, the likely relevant mechanism at play is that of neural persistence. Further investigations may pinpoint influences attributable to this mechanism by exploiting its characteristic sensitivity to masking (Pastukhov & Braun, 2013). Similarly, possible effects of the slower perceptual memory effects may be investigated using the long temporal gaps typically used to demonstrate it (Pearson & Brascamp, 2008).

What we have demonstrated with certainty is that there is a reliable effect of visual field location on primed awareness of SFM, supporting our stipulation that the divergence in perceptual experiences between fixated and peripheral vision extends to the integration of perceptual context. Given the results of Experiment 2, we are also confident that these effects are driven by differences in retinotopically organized parts of the visual system rather than higher order (non-retinotopic) processing.

To further characterize such effects and attempt to uncover their causes, future investigations might turn to stimulation dimensions that extend beyond those of the present study. Precision in SFM stimuli can be manipulated by altering the number of dots from which the object is composed (even at the same location); the strength of priming may be manipulated by altering dot contrast, influencing (dis)ambiguation of the prime; or the influence of acuity may be assessed by continuously manipulating stimulus eccentricity, as well as accounting for cortical magnification at different eccentricities by adjusting stimulus size.

The present study aimed to examine how a percept may be affected by location in the visual field; therefore, sphere diameter was kept constant for fixated and peripheral viewing, to avoid confounding location with size. However, adjusting stimulus size for cortical magnification may provide an avenue to revealing the underlying causes of the visual field effect. If differences in cortical representation account for it, then an increase of sphere diameter in the periphery, scaled to cortical magnification, may abolish it. To address this possibility, further research would have to overcome challenges related to sphere area, such as the potential influence of stimulation in the additional areas covered by larger peripheral stimuli (closer to fixation and/or farther from it). Further investigation may also address the influence of variations in acuity across the periphery of the visual field, such as the known horizontal–vertical anisotropy, whereby visual performance is better along the horizontal meridian than the vertical meridian, and the vertical meridian asymmetry, whereby visual performance is better along the lower than the upper vertical meridian (Barbot, Xue, & Carrasco, 2021).

Although we are not the first to use luminance disparity cues to disambiguate SFM displays (Freeman & Driver, 2006; Schwartz & Sperling, 1983), other recent research has used three-dimensional stereodisparity for this purpose (Schmack et al., 2016; Sterzer et al., 2008; Weilnhammer et al., 2017). Here, we aimed to examine the effect of fixated versus peripheral viewing under the assumption that sensory stimulation is encoded with less precision in the periphery. Therefore, we opted for using luminance disparity, which creates a strong perceptual bias without requiring stereopsis, which is known to decline with eccentricity (Ghahghaei, McKee, & Verghese, 2019; Siderov & Harwerth, 1995).Using stereodisparity would have made it impossible to tell whether the location effect was due to monocular encoding of the sensory stimulation or to further binocular integration, but it would be interesting to examine whether the present findings generalize to situations where disambiguation is achieved via three-dimensional cues.

 All of these options (and undoubtedly many others) deviate from the basic designs of the current study in fundamental ways but may provide appropriate avenues for exploring the effects seen here.

## Supplementary Material

Supplement 1
