# Supplementary material for: Fixation versus periphery in visual awareness: Differential effects of recent perceptual experience
Source: J Vis. 2025 Jun 3;25(7):2. doi: 10.1167/jov.25.7.2 (PMC12136115; doi:10.1167/jov.25.7.2)
Supplement: Supplement 1 [file jovi-25-7-2_s001.pdf]

Fixation versus periphery in visual awareness: Differential effects of immediate perceptual experience

Tim Gastrell, Matt Oxner, Frank Schumann, and David Carmel

Supplementary Materials

Section 1: Derivation of hypotheses from predictive coding theories of awareness

Experiment 1

We can illustrate the impact of a prior on bistable percept selection in the framework of predictive coding by plotting hypothetical prior (formed by initial viewing of the unambiguous sphere), likelihood (sensory evidence in the ambiguous sphere), and precision-weighted posterior distributions on an axis system where the x-axis represents the strength of sensory evidence for each interpretation during bistable viewing, and the y-axis represents probability (**Figure S1**). In this case, the mid-point of the x-axis represents the decision boundary between the two percepts. The bottom-up signal induced by an ambiguous SFM sphere can be thought of as a noisy competition between two populations of neurons – one favouring leftwards and the other favouring rightwards rotation, and the perceptual outcome is determined by a winner-takes-all competition between these two populations. Some models of bistable perception (e.g., Schmack et al., 2016; Weilnhammer et al., 2017, 2021) have thus represented the likelihood as a bimodal distribution, with identical peaks equally spaced on either side of the decision boundary. Such representations are useful for depicting the temporal dynamics that lead to perceptual transitions, via the accumulation of a prediction error that eventually updates the prior. Our illustration, however, aims to capture how precision affects the relative influences of the sensory information (likelihood) and prior on

the posterior at a specific moment – the ambiguous target's onset. For this purpose, a clear graphic depiction is provided by a different approach (Leptourgos et al., 2020) that uses a unimodal likelihood distribution representing the difference between the strengths of the sensory evidence for each percept in a momentary sampling (in our case, at stimulus onset).

Figure S1

Illustration of Bayesian Interactions Yielding Immediate Perception of an Unambiguous Sphere Following an Unambiguous Prime at the Same Retinal Location (Experiment 1)

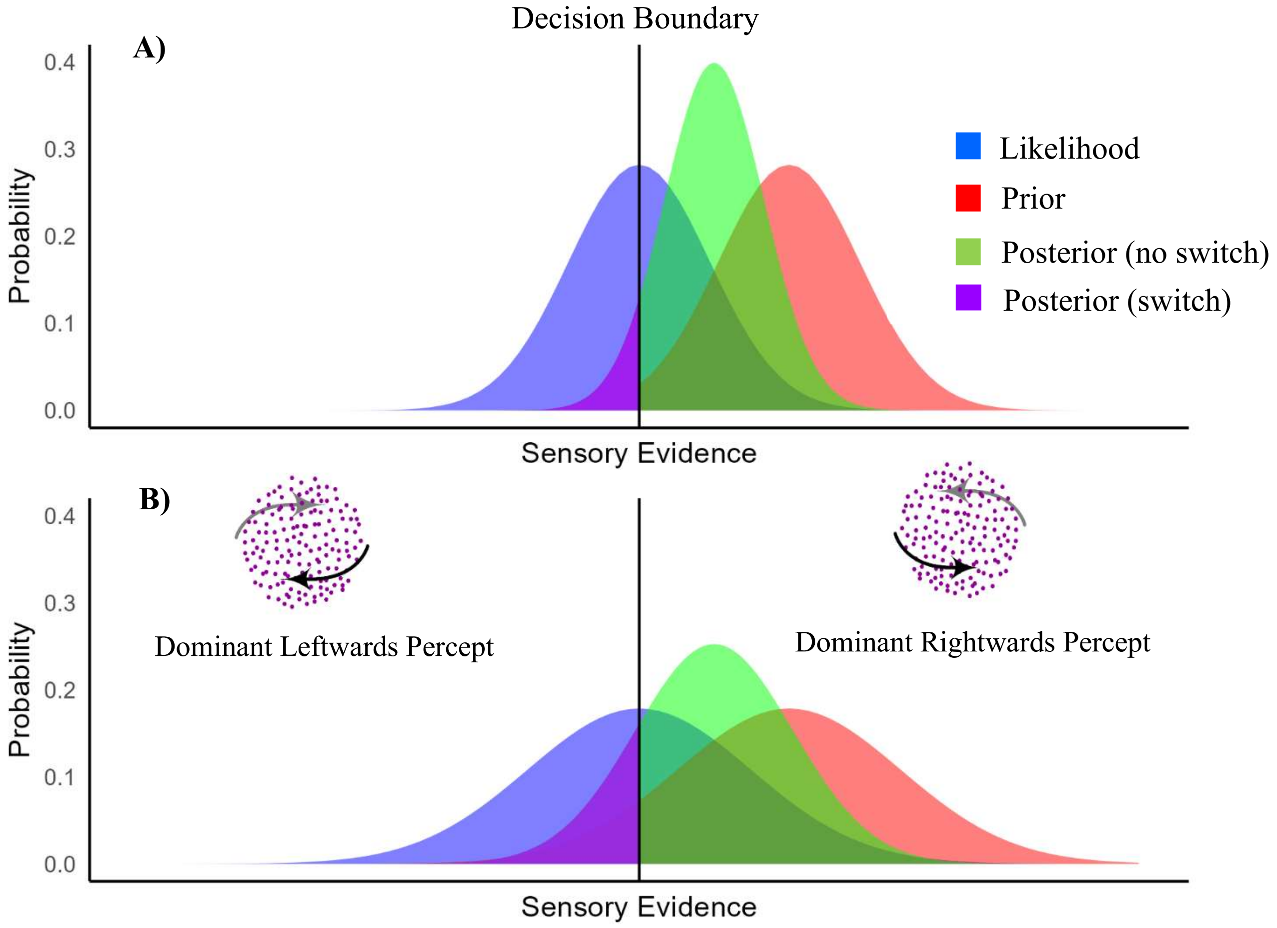

Note: Hypothetical distributions representing Bayesian likelihood (blue – centred at the decision boundary; black vertical), prior (red), and posterior (green/purple) probabilities for **(A)** high-precision foveal and (**B**) low precision peripheral encoding of the SFM sphere animation. Purple regions indicate the probability of perceiving a switch in rotation direction (a prior-inconsistent inference) between the unambiguous and ambiguous sphere phases.

This likelihood distribution straddles the mid-point of the axis system (blue distributions in **Figure S1**).

According to the Bayesian account, the prior probability of one interpretation is bolstered when ambiguous viewing follows an initial period of unambiguous viewing. In this case, a distribution representing the combined prior probability for each interpretation sits to one side of the mid-point, favouring the primed direction (red distribution in **Figure S1A**). The resulting posterior, representing the observer's momentary awareness of the ambiguous sphere, is biased towards the mean of the prior (for foveal vision, this is represented by the green region of the posterior distribution in **Figure S1A**); however, a portion of the posterior distribution (one tail – represented by the purple region in **Figure S1A**) occupies space on the other side of the midpoint. The area under this prior-inconsistent tail is thus the probability of a switch in rotation direction when the unambiguous sphere is replaced by the ambiguous one. When the spheres are presented to peripheral vision (**Figure S1B**), the prior and likelihood distributions have a low precision (reflected in large variance), leading to a posterior with a proportionally low precision and thus a higher probability of a switch (indicated by a larger purple region in **Figure S1B** compared to **Figure S1A**). Therefore, in Experiment 1 we predicted that if inference in bistable perception is precision weighted, observers should be more likely to report a switch when spheres are presented to peripheral, compared to foveal vision.

Experiment 2

In Experiment 2 the unambiguous prime and ambiguous target spheres were presented to different retinal locations. Presenting the unambiguous sphere to high-precision foveal vision (**Figure S2A**), establishes a precise prior for that interpretation (depicted by the red distribution). By contrast, following the sphere's movement to the periphery (during which it becomes ambiguous), the sensory evidence provides a relatively imprecise likelihood (depicted by the blue distribution). Precision-weighting predictive accounts propose that the precise prior should exert a heavy influence on the construction of posterior estimates (in green), pulling the mean away from the mid-point and leading to a small probability of a percept whose rotation is inconsistent with the prime (purple shaded tail of the posterior). Conversely, when the unambiguous prime is presented to peripheral vision (**Figure S2B**), the induced prior should be imprecise, while the subsequent foveally-presented ambiguous sphere provides a more precise likelihood. This leads to a posterior that, while still biased towards the primed direction, retains a comparatively higher probability of a prior-inconsistent percept (i.e., a switch).

Figure S2

Illustration of Bayesian Interactions Yielding Immediate Perception of an Unambiguous Sphere Following a Prime at a Different Retinal Location

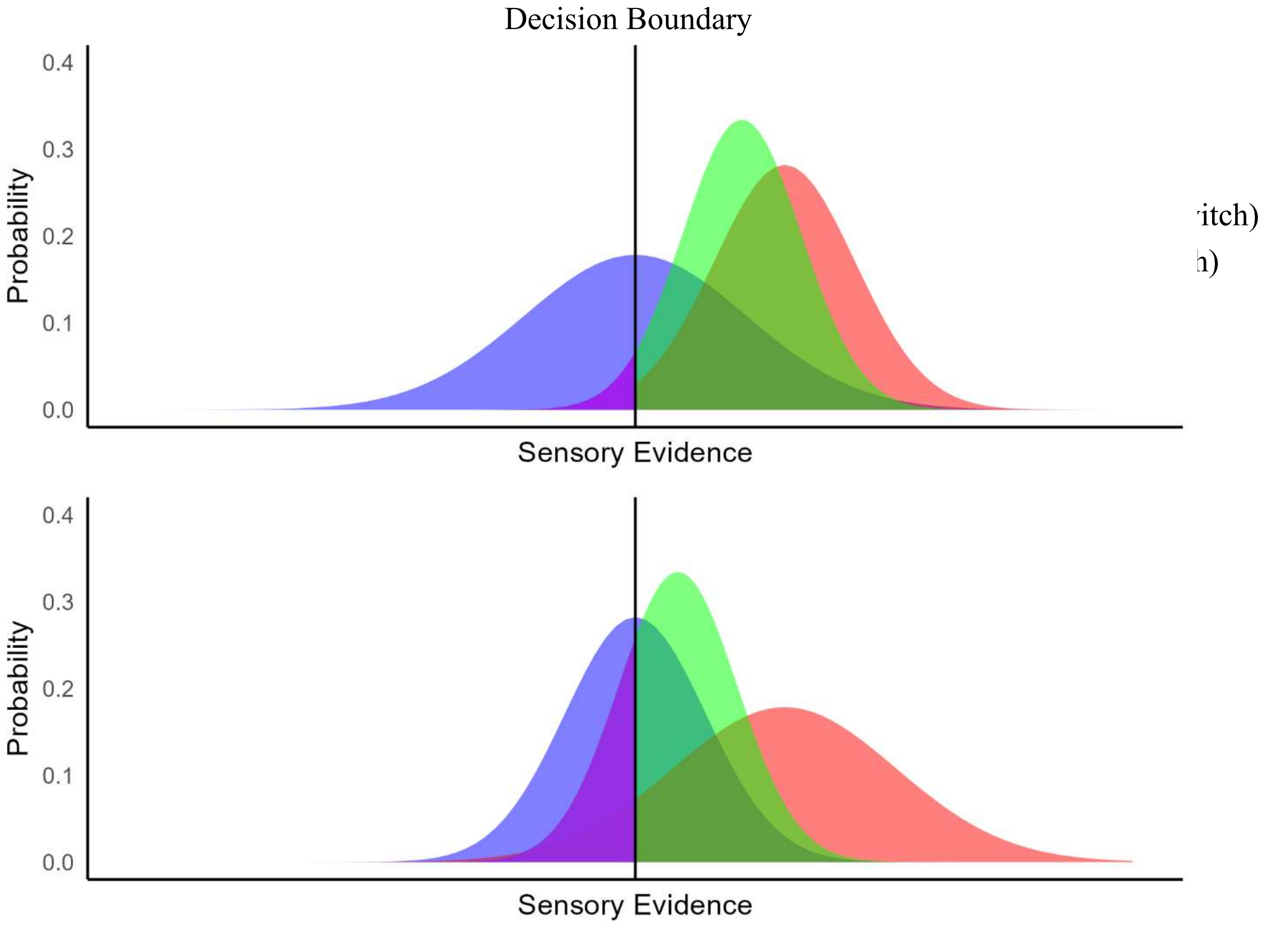

Note: Hypothetical distributions representing Bayesian likelihood (blue), prior (red) and posterior (green) probabilities for Experiment 2. Purple regions indicate the probability of perceiving a switch in rotation direction (a prior-inconsistent inference) between the unambiguous and ambiguous sphere phases. **A)** Prior encoded at fixation with high precision before ambiguous sphere is viewed in the periphery with low precision (low predicted switch probability). **B)** Prior encoded in the periphery with low precision before ambiguous sphere is view at fixation with high precision (high predicted switch probability).

References

Leptourgos, P., Bouttier, V., Jardri, R., & Denève, S. (2020). A functional theory of bistable perception based on dynamical circular inference. *PLOS Computational Biology*, *16*(12), e1008480. https://doi.org/10.1371/journal.pcbi.1008480

Schmack, K., Weilnhammer, V., Heinzle, J., Stephan, K. E., & Sterzer, P. (2016). Learning what to see in a changing world. *Frontiers in Human Neuroscience*, *10*(MAY2016), 1–12. https://doi.org/10.3389/fnhum.2016.00263

Weilnhammer, V., Fritsch, M., Chikermane, M., Eckert, A.-L., Kanthak, K., Stuke, H., Kaminski, J., & Sterzer, P. (2021). Evidence for an Active Role of Inferior Frontal Cortex in Conscious Experience. *Current Biology*, 1–13. https://doi.org/10.1101/2020.05.28.114645

Weilnhammer, V., Stuke, H., Hesselmann, G., Sterzer, P., & Schmack, K. (2017). A predictive coding account of bistable perception—A model-based fMRI study. *PLoS Computational Biology*, *13*(5), 1–21. https://doi.org/10.1371/journal.pcbi.1005536

Section 2: Preliminary analyses

Here, we detail preliminary analyses conducted before collapsing our measures across peripheral presentation side and sphere rotation axis in the four experiments. Unless otherwise specified, Greenhouse-Gesier corrected degrees of freedom and their corresponding p-values are reported where sphericity assumptions were violated.

Experiment 1

In our analysis of data collected in Experiment 1, we made comparisons between fixated and peripheral stimuli. In these analyses the peripheral measures were collapsed across trials presented to the left and to the right. Two tailed paired t-tests revealed no significant differences between the two peripheral locations in any condition (**Table 1**). Switch probabilities were thus collapsed into a single peripheral measure for the main analysis.

Table 1.

Experiment 1 Switch Probabilities for Peripheral Trials

Condition	Left Switch Probability Mean (SD)	Right Switch probability Mean (SD)	Two tailed paired t test
UA	0.69 (0.17)	0.67 (0.17)	$t(40) = 0.54, p = .589$
A	0.01 (0.02)	0.004 (0.02)	$t(40) = 0.12, p = .852$
UU	0.01 (0.09)	0.11 (0.10)	$t(40) = 0.51, p = .617$
US	0.89 (0.14)	0.91 (0.13)	$t(40) = 0.73, p = .465$

We also collapsed across the four possible rotation axes (45º, 135º, 225º, 315°; angles represent clockwise rotation from vertical). A repeated measures ANOVA was conducted for each of the four animation conditions (UA, UU, US, and A) comparing switch probabilities across the four axes as depicted in **Figure S5.** A significant main effect was found in the UA

condition, however pairwise post-hoc t-tests found no significant effects after Bonferroni correction for multiple comparisons.

Figure S3

Experiment 1 Switch Probabilities Across Sphere Rotation Axes and Condition

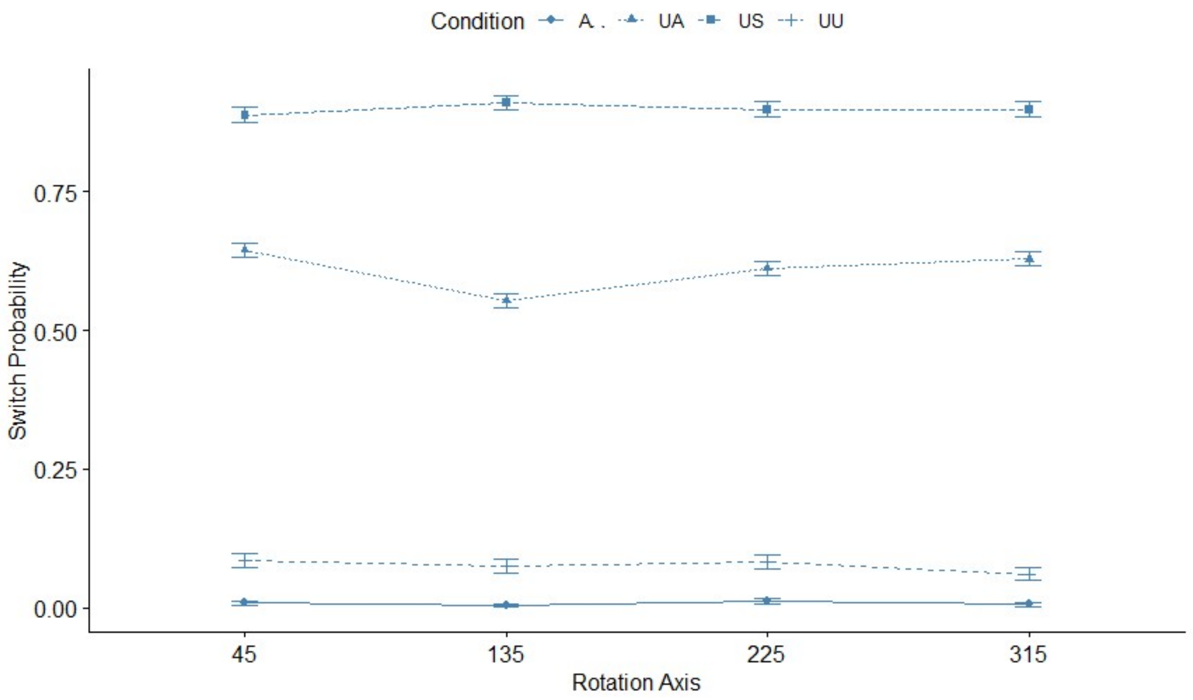

Note: Rotation axis in degrees. Error-bars indicate standard error.

Experiment 2

Like in Experiment 1, we collapsed across peripheral locations in our main analysis of Experiment 2. For this experiment, we collapsed across trials that started at fixation and moved into the left or right periphery and across trials that started in the left or right periphery and moved to the fixation point. To justify this treatment of the data, we conducted two-tailed paired t-tests comparing left and right peripheral locations within each of the four animation conditions (UA, A, UU, and US) for trials that started at fixation (**Table 2**) and for trials that started in the periphery (**Table 3**).

Only one significant effect was found after Bonferroni correction such that switch rates on US trials that started at fixation and moved into the left periphery were significantly greater than equivalent trials that started at fixation and moved to the right periphery (see **Table 2: US**). Given that this effect was restricted to the US condition, which was only used as a part of our exclusion criteria for this experiment, we believe it was appropriate to collapse across left and right peripheral locations in our main analysis.

Table 2.

*Experiment 2 Switch Probabilities for **Fixation to Periphery** Trials*

Condition	Fixation-Left Switch Probability Mean (SD)	Fixation-Right Switch Probability Mean (SD)	Two tailed paired t test
UA	0.45 (0.12)	0.47 (0.13)	$t(53) = 1.01, p = .316$
A	0.15 (0.19)	0.15 (0.17)	$t(53) = 0.26, p = .793$
UU	0.07 (0.09)	0.07 (0.09)	$t(53) = 0.13, p = .900$
US	0.92 (0.08)	0.88 (0.13)	$t(53) = 2.25, p = .029$ *

Table 3.

Experiment 2 Switch Probabilities for ***Periphery to Fixation*** *Trials*

Condition	Left-Fixation Probability Mean (SD)	Right-Fixation Probability Mean (SD)	Two tailed paired t test
UA	0.46 (0.11)	0.48 (0.11)	$t(53) = 0.76, p = .453$
A	0.19 (0.20)	0.18 (0.22)	$t(53) = 0.61, p = .543$
UU	0.07 (0.08)	0.09 (0.11)	$t(53) = 1.14, p = .261$
US	0.87 (0.12)	0.89 (0.12)	$t(53) = 0.79, p = .436$

Again, before collapsing across the four possible sphere rotation axes, we compared switch probabilities for each axis across each animation condition (**Figure S6).** Although there was a main effect of rotation axis on switch probability in the UA condition ($F(2.42, 193.26) = 3.03, p < .05, \eta_p^2 = 0.03$), post hoc pairwise comparisons revealed only one significant difference that withstood Bonferroni correction. Observers were significantly more likely to report switches in SFM spheres that rotated on 225° ($M = 0.53, SD = 0.20$) axes than 135° axes ($M = 0.41, SD = 0.21; t(53) = 2.75, p < .05$). Since this comparison was not significant in Experiment 1, we consider this difference likely to be a statistical anomaly that does not reflect a systematic effect, and thus collapsed across rotation axes in our main analyses for this experiment.

Figure S4

Experiment 2 Switch Probabilities Across Sphere Rotation Axes and Condition

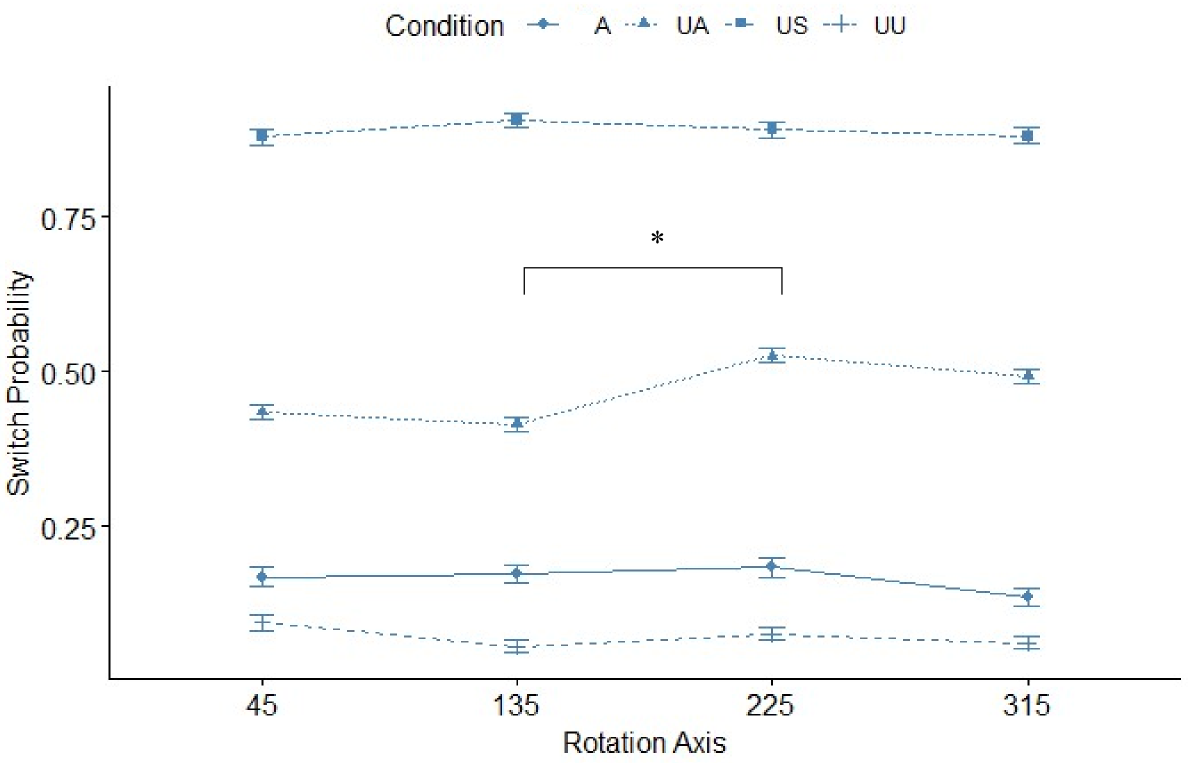

Note: Rotation axis in degrees. * Indicates post-hoc pairwise comparison reached statistical significance with $p < .05$ after Bonferroni correction for multiple comparisons. Error-bars indicate standard error.

Experiment 3

SFM task

Once again, we collapsed across left and right peripheral trials for our analysis of the SFM task. Two-tailed paired t-tests determined no significant differences between left and right peripheral trials in any of the four animation conditions (**Table 4**)

Table 4.

Experiment 3 Switch Probabilities for Peripheral Trials

Condition	Left Switch Probability Mean (SD)	Right Switch Probability Mean (SD)	Two tailed paired t test
UA	0.60 (0.14)	0.59 (0.13)	$t(80) = 0.86, p = .394$
A	0.08 (0.17)	0.08 (0.14)	$t(80) = 0.09, p = .927$
UU	0.09 (0.11)	0.09 (0.13)	$t(80) = 0.12, p = .905$
US	0.93 (0.10)	0.91 (0.11)	$t(80) = 1.31, p = .194$

We also collapsed across the four sphere rotation axes. Switch probabilities for each axis across each animation condition are plotted in **Figure S5**. The same analysis applied to rotation axes in Experiments 1 and 2 was applied here, revealing a significant main effect of rotation axis in the UA condition ($F(2.42, 211) = 3.07, p < .05, \eta_p^2 = 0.03$). Post-hoc pairwise comparisons revealed only one significant difference that withstood Bonferroni correction for multiple comparisons. Observers were significantly more likely to report switches in SFM spheres that rotated on 315° ($M = 0.53, SD = 0.20$) axes than 135° axes ($M = 0.41, SD = 0.21$) ($t(80) = 2.84, p < .05$).

Figure S5

Experiment 3 SFM Task Switch Probabilities Across Sphere Rotation Axes and Condition

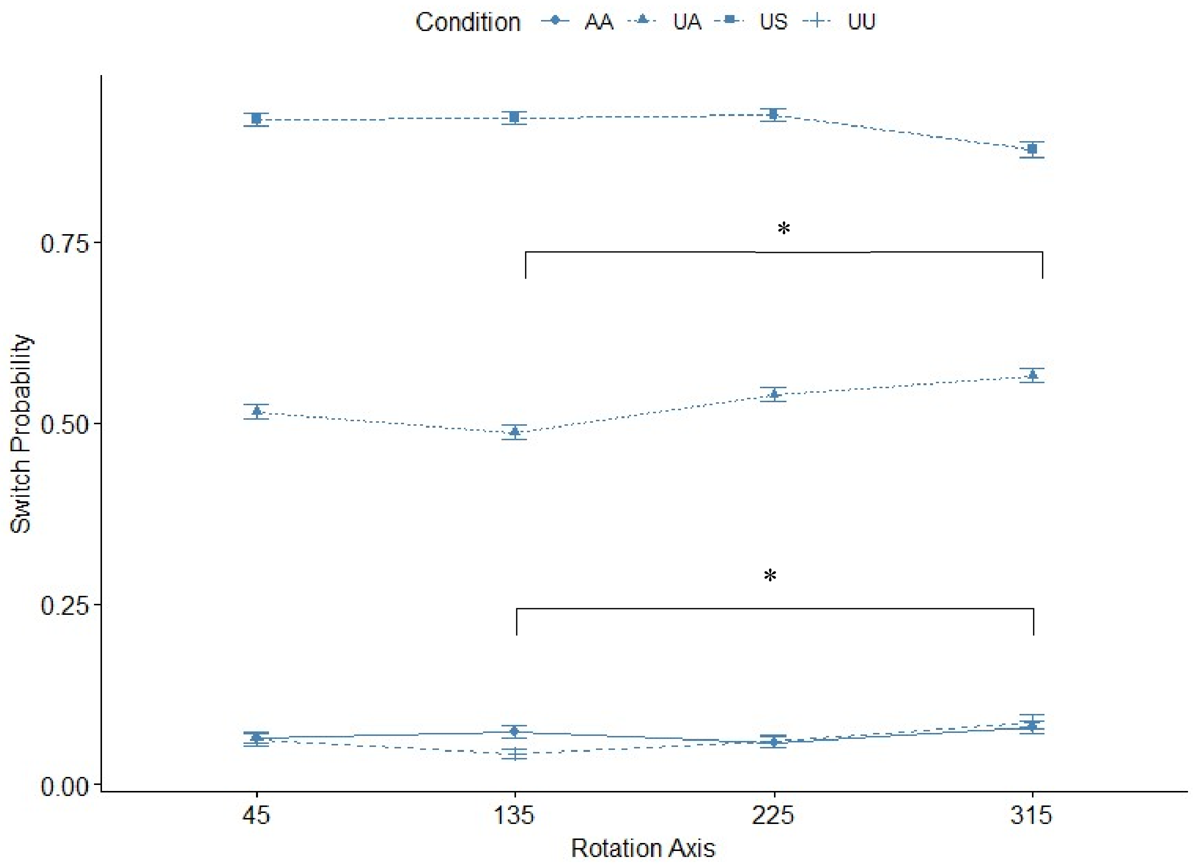

Note: Rotation axis in degrees. * Indicates post-hoc pairwise comparison reached statistical significance with $p < .05$ after Bonferroni correction for multiple comparisons. Error-bars indicate standard error.

We also observed a main effect of rotation axis on switch rates in the UU condition ($F(2.42, 211) = 4.80$, $p < .05$, $\eta^2 = 0.03$). Post hoc pairwise comparisons revealed only one significant difference that withstood Bonferroni correction for multiple comparisons. Observers were significantly more likely to report switches in SFM spheres that rotated on 315° ($M = 0.09$, $SD = 0.11$) axes than 135° axes ($M = 0.04$, $SD = 0.07$) ($t(80) = 3.10$, $p < .05$).

Since these comparisons were not significant in Experiments 1 and 2 (and occur for different angles than the one comparison that was significant in each of the previous

experiments), we consider these differences likely to be statistical anomalies that do not reflect systematic effects, and have collapsed across rotation axis in our main analyses for this task.

Experiment 4

Once again, we collapsed across left and right peripheral trials for our analysis of the SFM task. This time however, our preliminary analyses revealed that peripheral trials in the UA condition that were presented to the left hemifield elicited more switch reports compared to those presented to the right (**Table 5**). This pattern was also present in the other conditions but did not reach statistical significance.

Table 5.

Experiment 4 Switch Probabilities for Peripheral Trials

Condition	Left Switch Probability Mean (SD)	Right Switch Probability Mean (SD)	Two tailed paired t test
UA	0.67 (0.15)	0.64 (0.16)	$t(80) = 2.04, p = .04$
A	0.12 (0.04)	0.01 (0.03)	$t(80) = 1.00, p = .321$
UU	0.13 (0.14)	0.11 (0.12)	$t(80) = 1.13, p = .259$
US	0.91 (0.10)	0.89 (0.12)	$t(80) = 1.66, p = .100$

We therefore present separate analyses for left and right peripheral UA trials. Peripheral trials were equally split between left and right, with the overall number of peripheral trials being equal to that of fixated trials. This means there were only half as many trials in each peripheral location as at fixation; separate analysis of each side compared to fixation would necessitate comparisons between different numbers of trials. To circumvent this concern, we randomly split each participant's fixated trials into two subsets – one that was compared with left-peripheral trials, and one that was compared with right-peripheral trials. To ensure that any observed patterns were not due to a failure of randomisation, we

repeated this procedure three times, conducting the analysis for three different random-splits of the fixation trials. All three iterations are reported in **Table 6**.

Crucially, the effect of visual field location on switch probabilities (i.e., more switches in the periphery than at fixation) in the UA condition was present for both left and right peripheral trials, though the effect was stronger on the left.

Table 6.

Experiment 4 UA Analyses for Left and Right

Peripheral presentation mean (SD)	Fixated trial subset mean (SD)	Paired t-test (uncorrected)	Cohen's dz [95% CI]
Left: 0.67 (0.15)	A: 0.54 (0.24)	$t(80) = 4.49, p < .01$	$dz = 0.49$ [0.28, 0.75]
	B: 0.54 (0.24)	$t(80) = 4.49, p < .01$	$dz = 0.49$ [0.29, 0.74]
	C: 0.55 (0.25)	$t(80) = 4.25, p < .01$	$dz = 0.47$ [0.25, 0.74]
Right: 0.65 (0.16)	A: 0.55 (0.24)	$t(80) = 3.08, p < .01$	$dz = 0.34$ [0.14, 0.56]
	B: 0.55 (0.24)	$t(80) = 2.99, p < .01$	$dz = 0.33$ [0.13, 0.56]
	C: 0.55 (0.24)	$t(80) = 3.26, p < .01$	$dz = 0.36$ [0.16, 0.58]

We also systematically collapsed across the four possible sphere rotation axes. Switch probabilities for each axis across each animation condition are plotted in **Figure S6.** We applied the same analysis of rotation axes as in Experiments 1, 2 and 3, revealing a significant main effect of rotation in the UA condition $F(2.66, 212.55) = 7.46$, $p < .001$, $\eta_p^2 = 0.085$. Post hoc pairwise comparisons revealed three significant differences that withstood Bonferroni correction. Observers were significantly less likely to report switches in SFM spheres that rotated on 135° ($M = 0.55$, $SD = 0.50$) axes compared 45° ($M = 0.62$, $SD = 0.48$; $t(80) = 3.75$, $p < .01$), 225° ($M = 0.63$, $SD = 0.48$; $t(80) = 4.29$, $p < .01$) and 315° ($M = 0.62$, $SD = 0.49$; $t(80) = 2.80$, $p < .05$) axes.

Figure S6

Experiment 4 Switch Probabilities Across Sphere Rotation Axes and Condition

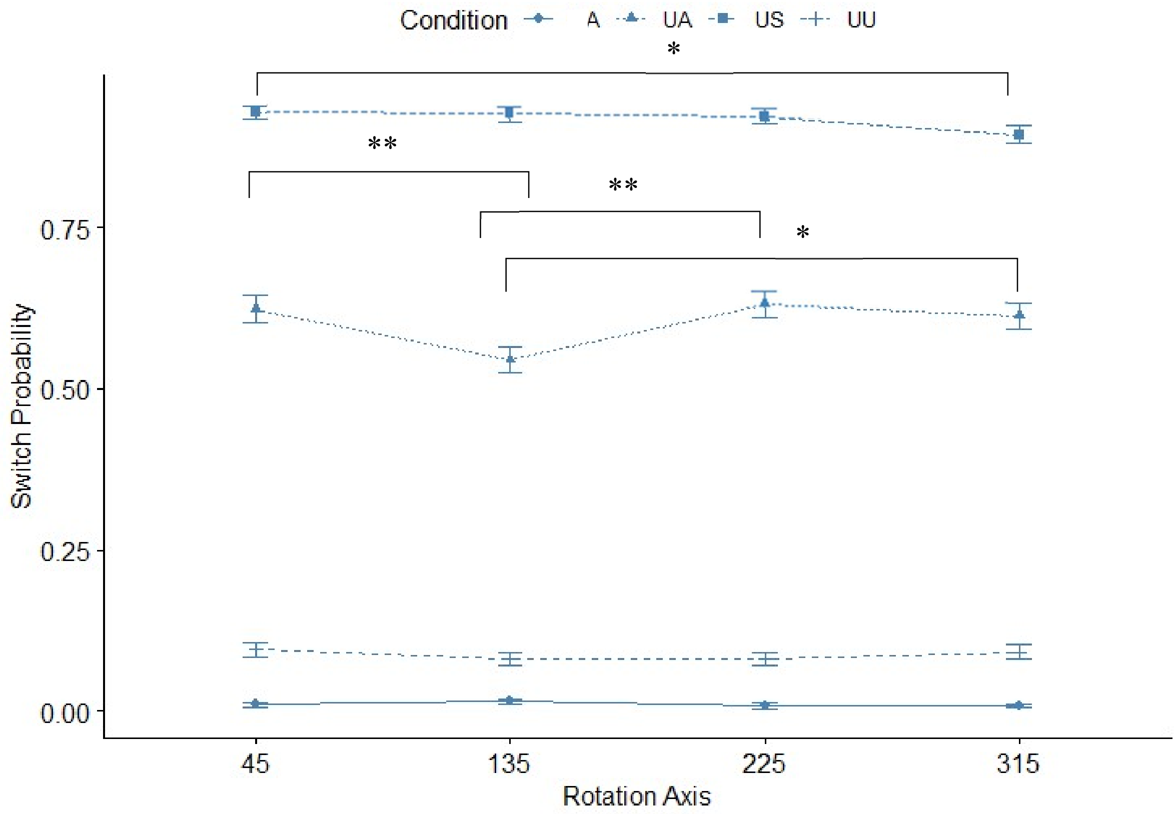

Note: Rotation axis in degrees. * Indicates post-hoc pairwise comparison reached statistical significance with $p < .05$ after Bonferroni correction for multiple comparisons. Similarly, ** indicates p < .01 and *** indicated p < .001. Error bars reflect the standard error.

A significant main effect of rotation axis was also found in the US condition (F(2.7, 215.86) = 2.96, $p < .05$, $\eta_p^2 = 0.036$). Post hoc pairwise comparisons revealed only one significant difference that withstood Bonferroni correction. Observers were significantly less likely to report switches for SFM spheres that rotated around 315° ($M = 0.89$, $SD = 0.31$) axes compared to 45° ($M = 0.93$, $SD = 0.26$; t(80) = 2.71, $p < .05$) axes.

Figure S7

Experiment 4 UA Switch Probabilities Across Sphere Rotation Axes and location

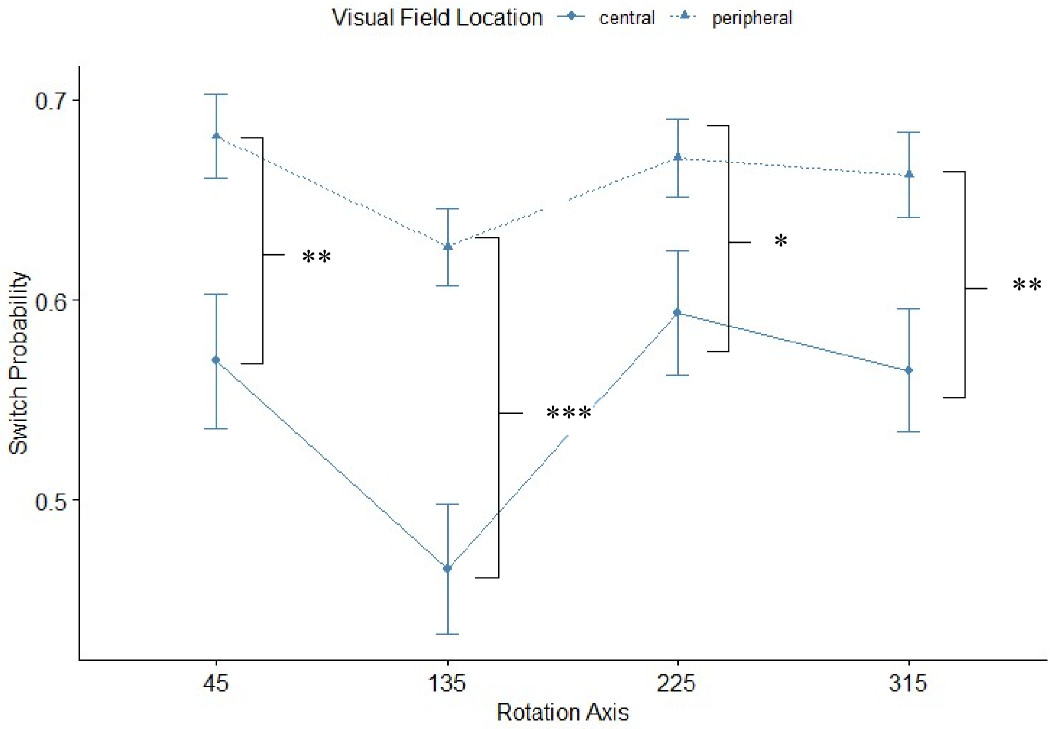

Note: Rotation axis in degrees. * Indicates post-hoc pairwise comparison reached statistical significance with $p < .05$ Similarly, ** indicates p < .01 and *** indicated p < .001. Error bars reflect the standard error.

Because the effects of rotation axis on switch reports in the UA condition include those we observed in Experiments 2 (135° vs. 225°) and 3 (135° vs 315°), we conducted a follow-up analysis to determine whether effects of rotation axis modulated the effect of location (fixation vs periphery) on switch reports. Mean switch reports across location and

rotation axis are presented in **Figure S7.** A 2 x 4 repeated measures ANOVA revealed no significant interaction between rotation axis and location ($F(2.74, 219.31) = 1.98$, $p = 0.123$, $\eta_p^2 = 0.024$). Follow-up comparisons indicated that peripheral trials elicit significantly more switch reports compared to fixation for all four of the rotation axes (**Table 7**).

Table 7.

Experiment 4 UA Analyses for Fixated versus Peripheral Trials at each Rotation Axis

Rotation Axis	Mean Switch Probability **Fixation** (SD)	Mean Switch Probability **Periphery** (SD)	Paired t test	Cohen's dz [95% CI]
45°	0.57 (0.50)	0.68 (0.47)	$t(80) = 2.93$, $p < .01$	$dz = 0.33$ [0.11 0.33]
135°	0.47 (0.50)	0.63 (0.48)	$t(80) = 4.56$, $p < .001$	$dz = 0.51$ [0.30 0.73]
225°	0.59 (0.49)	0.67 (0.47)	$t(80) = 2.31$, $p < .05$	$dz = 0.26$ [0.05 0.48]
315°	0.57 (0.57)	0.66 (0.47)	$t(80) = 2.86$, $p < .01$	$dz = 0.32$ [0.13 0.55]

Since rotation axis does not appear to significantly modulate the effect of location on switch rates in the UA condition, we collapsed across rotation axes in the main analysis.

Section 3: Motion Aftereffects (MAE) Task Details

Predictions

Figure S8 depicts the predicted pattern of points of subjective equality between two opposite motion signals in a perceptual nulling paradigm (Blake & Hiris, 1993). Without any adaptation, the PSE will be a random dot stimulus with no coherent motion signal: This is depicted by the dashed blue line, which indicates a hypothetical psychometric function (PMF) for perceived motion direction as a function of motion coherence in random-dot-kinematograms (RDKs) in the absence of a preceding adapter. Under adaptation (solid lines), however, the aftereffect diminishes observers' ability to detect the adapted direction, so a certain amount of coherent motion in the adapted direction is required for observers to be

Figure S8

Predicted Pattern of Motion Discrimination Results for MAE Task

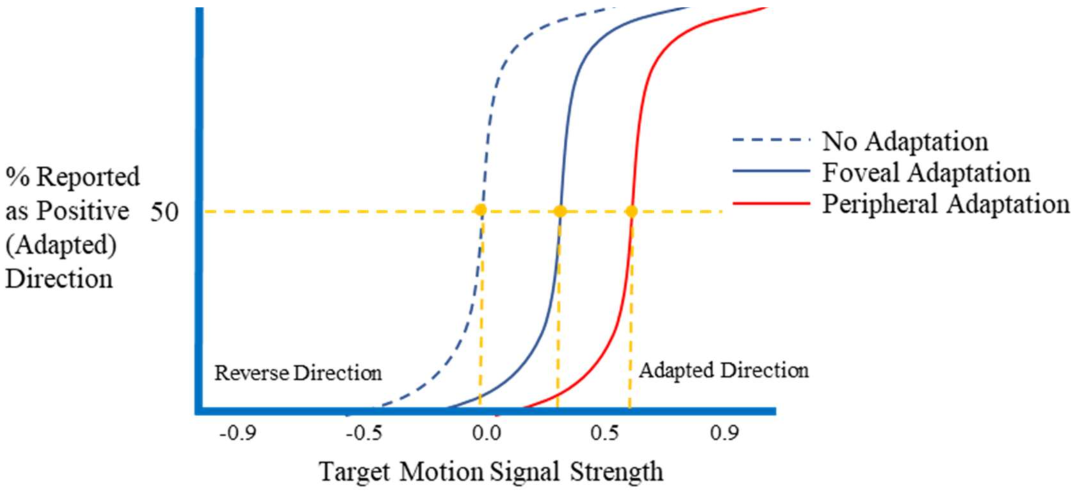

Note: Predicted pattern of psychometric functions (PMFs) for foveal (solid blue) and peripheral (solid red) trials in the MAE task if adaptation is stronger in the periphery. Yellow dots indicate points of subjective equality (PSEs), the signal strength at which an observer reports each direction with equal probability.

equally likely to report either direction. The stronger the MAE, the more the PSE will be shifted in the adapted (positive) direction.

Design of Random-Dot-Kinematogram Stimuli

The targets were RDK stimuli, similar to the classic dot-motion targets used to study motion processing (Blake & Hiris, 1993; Britten et al., 1992; Castet et al., 2002). Each RDK target contained 150 grey dots, randomly positioned within a circular aperture (radius = 1.0 dva), whose properties were identical to those used to compose the ambiguous SFM sphere in Experiments 1 and 2. We had two reasons for using 150 dots for the targets instead of the 300 dots used for the unambiguous sphere adapters: First, the bright dots which are typically seen as the 'front' surface of an unambiguous sphere – and thus whose motion typically corresponds to the perceived rotation direction – are only ever half of the dots in the entire animation. Second, informal observations during piloting suggested that using 300 dots in the target stimulus resulted in an animation with a much higher perceived dot density than that of the ambiguous SFM animation used in previous experiments. We believe this was a result of the different types of motion used in each stimulus (dots in SFM spheres followed a sinusoidal motion profile to indicate depth, whereas dots in an RDK have uniform velocity which indicates a single surface). When only 150 dots were used in the RDK target stimulus, the dot density experienced was qualitatively much closer to that of the ambiguous SFM sphere it was designed to emulate.

A coherent motion signal was added to each RDK target by randomly selecting a fixed percentage of dots to move in the signal direction between each pair of consecutive frames while the remaining dots moved in random directions (**Figure S9A**). Importantly, this means a *new* set of dots was selected every with frame transition. We selected this method to diminish the probability (especially for weaker motion signals) that observers could make accurate discriminations by focussing on the motion of a single dot, rather than the entire display. The strength of the motion signal in each target was manipulated by increasing or decreasing the percentage of dots selected to move in the signal direction.

Because the component dots of SFM spheres follow a sinusoidal speed profile, they move slowly near the edges of the sphere and quickly near the centre. This means that any motion adaptation induced by the unambiguous sphere stimulus is strongest in the centre of the sphere, and weakest towards the edges. This could have enabled participants to correctly perceive motion in the MAE task by attending to motion at the edge of the target, despite stronger aftereffects precluding accurate judgement at the centre. To prevent participants using this kind of strategy we made the radius of the aperture through which the RDK target was visible 13% smaller (0.87 dva radius) than that of the SFM adapter stimulus (**Figure S9B**). This aperture radius was determined as the full-width-half-maximum of a unit sinusoid (0.87), meaning that the area of the target visible to participants was the area that received the most motion adaptation (this is where the component dots of the unambiguous sphere adapter were between 50-100% of their maximum speed).

The reduced aperture size relative to the adapter was achieved using an occluding annulus of the same colour and luminance as the background, whose aperture was 0.87 dva in radius. Thus, while the dots were generated within a 1.0 dva radius circular area, they were only visible within the 0.87 dva radius aperture. This detail is important to note because it meant that the density of dots within the target was the same as that of a target presented without the occluding annulus.

To account for the restricted area within which motion adaptation could influence judgements, target dot speed was set to 0.73 dva/s, which is equal to the average dot speed within the equivalent area of the unambiguous sphere adapter. Dots that moved beyond 1.0 dva from the centre of the RDK reappeared (i.e., ‘wrapped around’) at random new positions along this boundary, restricted to the side of the circle opposite to that which signal dots travel towards (see **Figure S9C** for illustrative examples).

Figure S9

Schematic Depictions of Random-Dot-Kinematogram (RDK) Target Stimuli

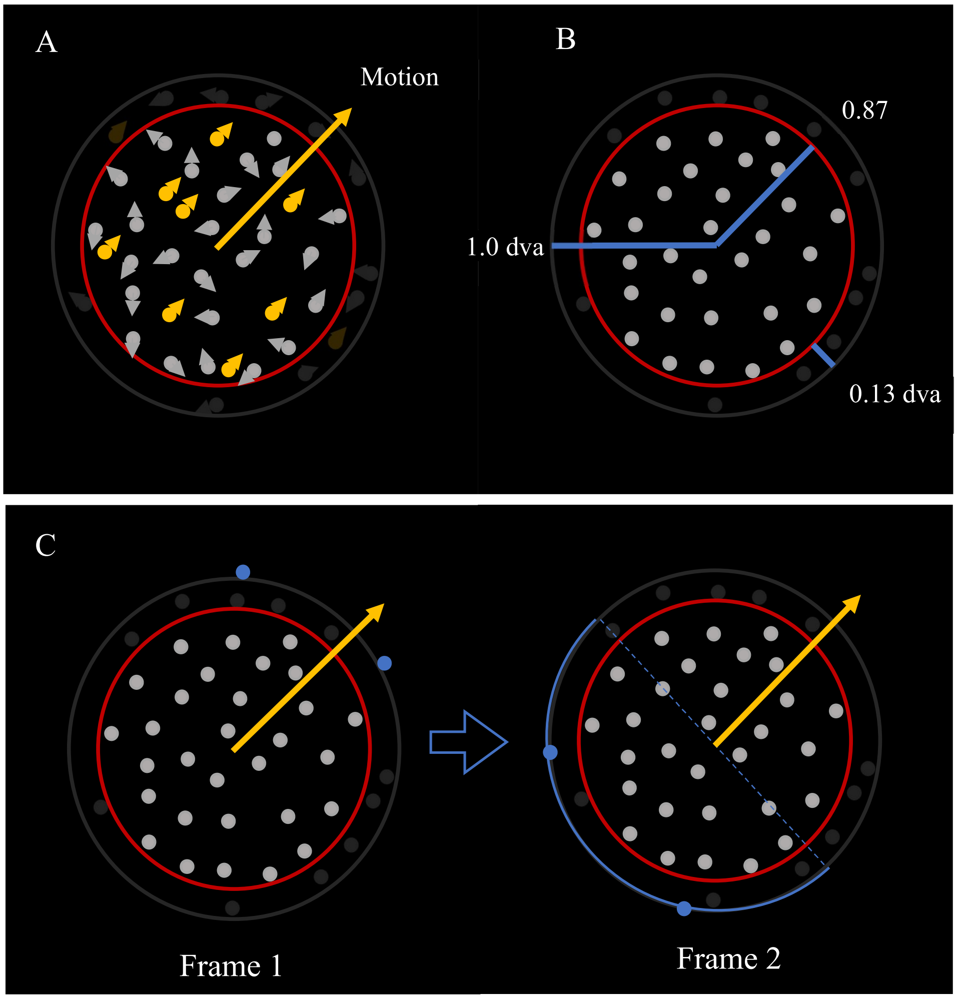

Note: Schematic depictions of RDK target stimuli. **A)** in each transition from one frame to the next, dots from the first frame are randomly selected to move a fixed distance in the motion signal direction (yellow dots) while the remaining dots move the same distance in random directions. **B)** Dots are randomly generated and move in a circular area with a 1.0 dva radius represented by the grey, outer circle. Only dots within the red circle are visible to observers; dots occupying the space between the red and grey circles (shaded) are not visible to the observer. **C)** Dots that move beyond the outer boundary (blue dots in Frame 1) are 'wrapped-around' – i.e., repositioned at random locations on the side of the circle (blue arc in Frame 2) opposite to the motion signal angle (yellow arrow).

Exclusion Criteria (Experiment 3)

In line with our pre-registered criteria, in addition to low catch-trial performance in the SFM task (which led to the exclusion of 14 participants from analysis), participants were also excluded from analysis for the following reasons: First, if they did not mainatin fixation and/or did not respond to more than 33% of the total trial count in either the SFM of MAE task. Eye movements were determined using the same criteria as Experiment 2. Three participants were excluded from analysis on this basis. Second, we exlcuded participants who did not reliably report the correct motion direction on trials with 0.9 coherence (i.e., 90% of the dots moved uniformly in the signal direction) in the MAE task. Participants who reported the incorrect direction on more than 30% of 0.9 coherence trials in any one of the two directions at any one of the visual field locations (fixation or periphery) were exlcuded. Two participants were exlcuded from analysis on this basis.

References

Blake, R., & Hiris, E. (1993). Another means for measuring the motion aftereffect. *Vision Research*, *33*(11), 1589–1592. https://doi.org/10.1016/0042-6989(93)90152-M

Britten, K., Shadlen, M., Newsome, W., & Movshon, J. (1992). The analysis of visual motion: A comparison of neuronal and psychophysical performance. *The Journal of Neuroscience*, *12*(12), 4745–4765. https://doi.org/10.1523/JNEUROSCI.12-12-04745.1992

Castet, E., Keeble, D. R. T., & Verstraten, F. A. J. (2002). Nulling the motion aftereffect with dynamic random-dot stimuli: Limitations and implications. *Journal of Vision*, *2*(4), 302–311. https://doi.org/10.1167/2.4.3

Section 4: Supplementary Figure for Experiment 4

Figure S10

Experiment 4: Comparison of BCEA across location and Participant Response

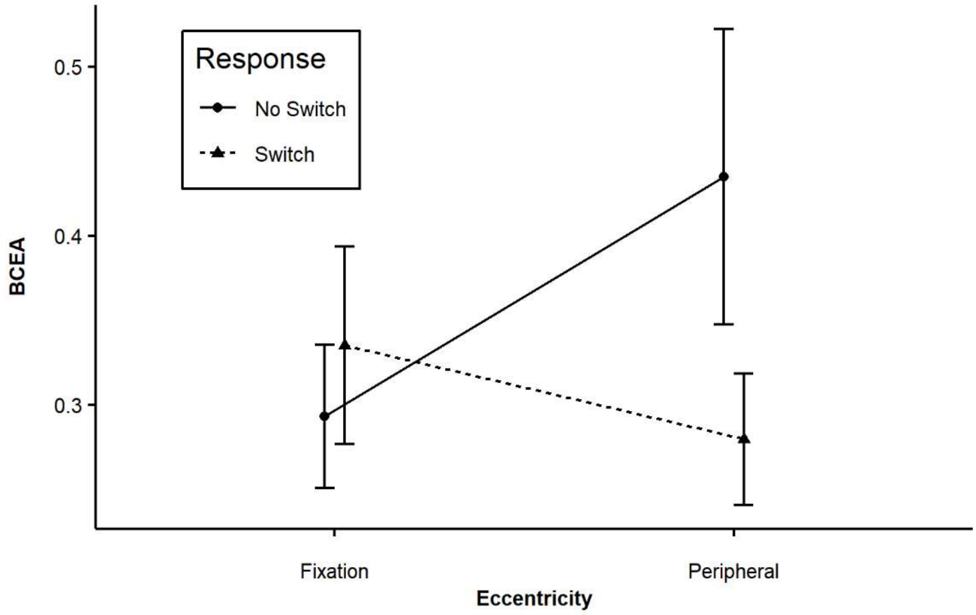

Note: Error bars depict the standard error.

Section 5: Mixed-Effects Model Outputs

All models were fit using version 1.1-35.5 the “lme4" package in R.

Experiment 1

Logistic Mixed Effects Model:

Response (switch no = 0, yes = 1) ~ Eccentricity (fixation = 0, periphery = 1) + (1 | ID):

**Condition**	Predictor	Z	Beta (OR)	95% CI [low, high]	p-value
**UA**	Intercept	1.63	0.18 (1.20)	[-0.04, 0.41]	.104
	Eccentricity	11.27	0.65 (1.92)	[0.54, 0.76]	< .001
**UU**	Intercept	-16.33	-3.07 (0.05)	[-3.44, -2.70]	< .001
	Eccentricity	3.90	0.70 (2.01)	[0.35, 1.05]	< .001
**US**	Intercept	13.40	2.48 (11.94)	[2.11, 2.84]	< .001
	Eccentricity	-0.12	-0.02 (0.98)	[-0.32, 0.28]	.905
**A**	Intercept	-9.26	-4.65 (0.01)	[-5.64, -3.67]	< .001
	Eccentricity	-1.63	-0.95 (0.39)	[-2.10, 0.20]	.104

Baselined UA (Participant Level): Linear Mixed Effects Model

Proportion Switch Response ~ Eccentricity (fixation = 0, periphery = 1) + (1 | ID):

Intercept (Beta = 0.48, 95% CI [0.41, 0.54], t(78) = 14.99, p < .001)

Eccentricity (beta = 0.10, 95% CI [0.01, 0.18], t(78) = 2.35, p = 0.022; Std. beta = 0.46, 95% CI [0.07, 0.85])

Experiment 2

Logistic Mixed Effects Model:

Response (switch no = 0, yes = 1) ~ Eccentricity (fixation = 0, periphery = 1) + (1 | ID):

**Condition**	Predictor	Z	Beta (OR)	95% CI [low, high]	p-value
**UA**	Intercept	-2.72	-0.12 (0.89)	[-0.23, -0.01]	.030
	Eccentricity	-0.66	-0.03 (0.97)	[-0.12, 0.06]	.508
**UU**	Intercept	-18.47	-2.68 (0.07)	[-2.96, -2.39]	< .001
	Eccentricity	-0.46	-0.07 (0.93)	[-0.38, 0.24]	.648
**US**	Intercept	15.59	2.16 (8.67)	[1.89, 2.43]	< .001
	Eccentricity	1.8	0.23 (1.26)	[-0.02, 0.49]	.072
**A**	Intercept	-9.09	-2.08 (0.12)	[-2.52, -1.63]	< .001
	Eccentricity	-2.80	-0.33 (0.72)	[-0.57, -0.10]	.005

Baselined UA (Participant Level): Linear Mixed Effects Model

Proportion Switch Response ~ Eccentricity (fixation = 0, periphery = 1) + (1 | ID):

Intercept (Beta = 0.21, 95% CI [0.16, 0.27], $t(104) = 7.77$, $p < .001$)

End Eccentricity (beta = 0.03, 95% CI [-0.02, 0.08], $t(104) = 1.30$, $p = 0.197$; Std. beta = 0.16, 95% CI [-0.09,0.42])

Experiment 3

Logistic Mixed Effects Model:

Response (switch no = 0, yes = 1) ~ Eccentricity (fixation = 0, periphery = 1) + (1 | ID):

**Condition**	Predictor	Z	Beta (OR)	95% CI [low, high]	p-value
**UA**	Intercept	-3.33	-0.16 (0.85)	[-0.26, -0.07]	< .001
	Eccentricity	14.422	0.57 (1.77)	[0.49, 0.65]	< .001
**UU**	Intercept	-21.62	-3.51 (0.03)	[-3.83, -3.19]	< .001
	Eccentricity	5.95	0.87 (2.39)	[0.58, 1.15]	< .001
**US**	Intercept	18.63	2.54 (12.68)	[2.27, 2.80]	< .001
	Eccentricity	1.67	0.20 (1.22)	[-0.04, 0.43]	.096
**A**	Intercept	-12.33	-4.41 (0.01)	[-5.11, -3.71]	<. 001
	Eccentricity	1.67	0.26 (1.30)	[-0.04, 0.55]	.095

Baselined UA (Participant Level): Linear Mixed Effects Model

Proportion Switch Response ~ Eccentricity (fixation = 0, periphery = 1) + (1 | ID):

Intercept (Beta = 0.36 (95% CI [0.31, 0.40], t(158) = 15.73, p < .001)

Eccentricity (beta = 0.08, 95% CI [0.04, 0.12], t(158) = 3.81, p < .001; Std. beta = 0.38, 95% CI [0.18, 0.57])

PSE (Participant Level): Linear Mixed Effects Model

PSE ~ Eccentricity (fixation = 0, periphery = 1) + (1 | ID):

Intercept (Beta = 0.07, 95% CI [0.05, 0.09], t(158) = 6.61, p < .001)

Eccentricity (beta = 0.06, 95% CI [0.04, 0.08], t(158) = 5.30, p <.001; Std. beta = 0.58, 95% CI [0.36, 0.79])

Trial level UA Responses with participant level PSE as covariate:

**Model**	Predictor	Z	Std. Beta (OR)	95% CI [low, high]	p-value
**UA response ~ Eccentricity * PSE + (1|ID)**	Intercept	-3.88	-0.21 (0.81)	[-0.32, -0.11]	< .001
	Eccentricity	8.47	0.55 (1.73)	[0.46, 0.63]	< .001
	PSE	1.94	0.07 (1.07)	[-8.63e-04, 0.14]	.053
	Eccentricity * PSE	-1.03	-0.05 (0.95)	[-1.50, 0.46]	.301
**UA response ~ Eccentricity * Δ PSE + (1|ID)**	Intercept	-2.51	-0.12	[-0.23, -0.01]	.031
	Eccentricity	11.98	0.57	[0.49, 0.65]	< .001
	Δ PSE	-1.46	-0.07	[-0.17, 0.02]	.146
	Eccentricity * PSE	0.917	0.04	[-0.04, 0.11]	.359

Experiment 4

Logistic Mixed Effects Model:

Response (switch no = 0, yes = 1) ~ Eccentricity (fixation = 0, periphery = 1) + (1 | ID):

**Condition**	Predictor	Z	Beta (OR)	95% CI [low, high]	p-value
**UA**	Intercept	2.84	0.21 (1.23)	[0.07, 0.36]	.005
	Eccentricity	12.73	0.51 (1.67)	[0.43, 0.59]	< .001
**UU**	Intercept	-21.82	-3.03 (0.05)	[-3.30, -2.76]	< .001
	Eccentricity	6.49	0.79 (2.20)	[0.55, 1.02]	< .001
**US**	Intercept	20.91	2.95 (19.12)	[2.67, 3.23]	< .001
	Eccentricity	-3.84	-0.46 (0.63)	[-0.70, -0.23]	< .001
**A**	Intercept	-12.99	-5.22 (0.01)	[-6.01, -4.43]	< .001
	Eccentricity	-0.507	-0.17 (0.84)	[-0.83, 0.49]	.612

Baselined UA (Participant Level): Linear Mixed Effects Model

Proportion Switch Response ~ Eccentricity (fixation = 0, periphery = 1) + (1 | ID):

Intercept (Beta = 0.48 (95% CI [0.43, 0.52], t(158) = 20.19, p < .001)

Eccentricity (Beta = 0.06, 95% CI [-8.21e-03, 0.12], t(158) = 1.72, p = 0.087; Std. beta = 0.26, 95% CI [-0.04, 0.56])

BCEA (Participant Level): Linear Mixed Effects Model

BCEA ~ Eccentricity (fixation = 0, periphery = 1) + (1 | ID):

Intercept (Beta = 0.35 (95% CI [0.26, 0.43], t(157) = 8.16, p < .001)

Eccentricity (beta = -0.02, 95% CI [-0.12, 0.08], t(157) =-0.39, p = 0.697; Std. beta = -0.05, 95% CI [-0.31, 0.21])

BCEA (Trial Level): Linear Mixed Effects Model

BCEA ~ Eccentricity (fixation = 0, periphery = 1) + (1 | ID):

Intercept (Beta 0.54 (95% CI [0.39, 0.69], t(9220) = 7.18, p < .001)

Eccentricity (beta = -0.11, 95% CI [-0.29, 0.06], t(9220) = -1.25, p = 0.210; Std. beta = -0.03, 95% CI [-0.07, 0.01])

UA responses with BCEA as covariate:

**Model**	Predictor	Z	Std. Beta (OR)	95% CI [low, high]	p-value
**UA response ~ Eccentricity * BCEA + (1|ID)**	Intercept	2.46	0.19	[0.04, 0.34]	.014
	Eccentricity	12.06	0.54	[0.45, 0.63]	< .001
	BCEA	0.78	0.02	[-0.04, 0.08]	.437
	Eccentricity * BCEA	-0.80	-0.04	[-0.13, 0.06]	.426
